# Interpretable Topic Modeling of Spontaneous Speech in Depression Using Large Language Models: Multilingual Four-Cohort Study

**DOI:** 10.2196/99185

**Published:** 2026-08-17

**Authors:** Gustave Cortal, Sélim Benjamin Guessoum, Xuan-Nga Cao, Santiago de Leon-Martinez, Enrique Baca-García, Rachid Riad

**Affiliations:** 1 Callyope, Pépinière d'entreprises Paris Santé Cochin, 29 Rue du Faubourg Saint-Jacques, Paris, 75014, France, 33 0666522141; 2 Université Paris-Saclay, Centre de recherche en Epidémiologie et Santé des Populations, Villejuif, France; 3 Brno University of Technology, Brno, Czech Republic; 4 Kempelen Institute of Intelligent Technologies, Bratislava, Slovakia; 5 Department of Psychiatry, Hospital Universitario Fundación Jiménez Díaz, Madrid, Madrid, Spain; 6 Department of Psychiatry, University Hospital Rey Juan Carlos, Mostoles, Spain; 7 Department of Psychiatry, Hospital Universitario General de Villalba, Madrid, Madrid, Spain; 8 Department of Psychiatry, University Hospital Infanta Elena, Valdemoro, Spain; 9 Department of Psychology, Catholic University of the Maule, Talca, Maule Region, Chile; 10 Department of Psychiatry, Universidad Autónoma de Madrid, Madrid, Madrid, Spain; 11 Centro de Investigación Biomédica en Red de Salud Mental, Madrid, Madrid, Spain

**Keywords:** depression, thematic analysis, speech analysis, large language models, topic modeling, natural language processing, mental health, computational psychiatry

## Abstract

**Background:**

Depression is underdiagnosed worldwide, and clinicians rely on interpreting patients’ subjective speech. Qualitative analysis of patient language does not scale, and existing computational approaches describe topics with keyword lists that miss clinical nuance.

**Objective:**

We evaluated whether clustering spontaneous speech transcripts with large language models (LLMs) yields clusters whose membership is associated with validated clinical scales across multilingual cohorts, and whether LLMs can render those clusters human-readable through fine-grained natural-language descriptions. We further examined which interview questions yield clusters most strongly associated with clinical status, and how sociodemographic factors relate to cluster membership.

**Methods:**

We analyzed spontaneous speech transcripts from 4 independent cohorts: a French general population sample (1338 participants) and 3 clinical samples in Italian (n=116), Chinese (n=52), and Spanish (n=90). Responses to open-ended questions were transcribed, embedded with a multilingual language model, dimensionally reduced, and grouped by density-based clustering. An LLM then summarized each cluster into a natural-language description. Cluster membership was tested for association with validated clinical scales (Patient Health Questionnaire-9, Beck Depression Inventory, Generalized Anxiety Disorder 7-item scale, Athens Insomnia Scale, Multidimensional Fatigue Inventory, and Columbia Suicide Severity Rating Scale), clinician-assigned depression diagnoses, and sociodemographic factors (age, education, and sex).

**Results:**

Unsupervised clustering yielded clusters significantly associated with clinical scores in the French, Italian, and Chinese cohorts, with an exploratory association in the smaller Spanish cohort. In the French general population, Patient Health Questionnaire-9 depression scores differed across clusters (η²=0.19, 95% CI 0.17 to 0.24, *P*<.001), as did anxiety (Generalized Anxiety Disorder 7-item scale), insomnia (Athens Insomnia Scale), and fatigue (Multidimensional Fatigue Inventory) scores (η²=0.14 to 0.16, all *P*<.001). In the clinical cohorts, cluster membership was associated with clinician-diagnosed depression in the Italian sample (Cramér *V*=0.74, 0.65 to 0.85, *P*<.001) and with major depressive disorder diagnosis in the Chinese sample (*V*=0.55, 0.36 to 0.78, *P*=.001). A suicide-risk association in the smaller Spanish sample (Columbia Suicide Severity Rating Scale) was unstable and is reported as exploratory (mean *V*=0.27). The feelings-and-sleep question yielded the strongest clinical associations, whereas questions about past or future events yielded small effect sizes (η²≤0.05). Age (η²=0.27, 0.24 to 0.32) and sex (Cramér *V*=0.22, 0.21 to 0.30) were each associated with cluster membership for the “describe your last 24 hours” question (both *P*<.001).

**Conclusions:**

Fine-grained LLM-based topic modeling automates the labor-intensive early stages of thematic analysis, in minutes of compute, and recovers core psychiatric constructs across languages. It produces human-readable cluster descriptions. Certain interview questions yield clusters more strongly associated with clinical status than others, and sociodemographic factors shape topic content independently of clinical status, an often-overlooked confound. As the cohorts differ, cross-cohort observations are preliminary without generalizability. Screening, treatment-planning, and decision-support uses remain to be established prospectively.

## Introduction

Depressive disorders are among the major challenges in psychiatry. These highly prevalent conditions are estimated to affect 30% to 40% of individuals during their life, across genders and regions worldwide [1]. Depression is a leading contributor to global disability and affects functioning and mortality. Depression remains underdiagnosed and undertreated worldwide [2].

Psychiatrists face distinct, complex assessment challenges in comparison to other medical specialties. Most medical fields rely on complementary investigations and paraclinical tests to assess the objective status of patients. Psychiatrists’ evaluation depends on interpreting patients’ subjective experiences in their own words, then mapping these diverse narratives onto standardized *DSM* (*Diagnostic and Statistical Manual of Mental Disorders*) criteria [3]. This “transformation” task, from individual expressions of suffering to fixed sets of symptoms and diagnoses, yields variable and modest interrater reliability [4,5]. Moreover, current standardized tools in psychiatry consist of rating scales (self-reported and clinician-administered) that rely on pre-established constructs [6,7]. Even when useful, they do not capture the full complexity of the lived experience of affective disorders [8].

This modest interrater reliability is not due to inadequate clinical practice, but rather the inherent complexity of psychiatric phenomena and the current limitations of validated assessment tools. In 2021, while cardiology has over 700 US Food and Drug Administration–approved devices and neurology over 300 devices to support clinical decision-making, psychiatry has fewer than 20 devices [9], and no AI-powered devices have been adopted despite AI’s integration across other specialties [10]. This technology gap is particularly surprising given that patient language, the primary data source in psychiatric consultations, is rarely analyzed in a systematic way despite extensive evidence linking linguistic patterns to mood disorder symptomatology [11-13].

Language analysis and interpretation, or the qualitative analysis of patients’ speech content, is a central part of clinical practice. It helps clinicians understand patients’ narratives about themselves, their lives, and their illnesses, and it forms the basis of psychotherapy. However, producing reliable data on this topic is highly resource-intensive, as shown in a prior study [14]. This study required extensive collaborative workshops and global expert panels to identify how depression manifests through specific linguistic themes such as altered temporal experience, embodied distress, and existential emptiness. There is also a prognostic value when these themes are extracted: how patients narrate autobiographical memories predicts depression trajectory, with overgeneral vs specific memory styles serving as age-dependent markers [15]. Natural language processing has been used to analyze such narrative features automatically, classifying emotions in personal narratives from their underlying psychological components [16] and relating stylistic patterns to psychological states [17]. Yet, clinicians struggle to analyze and interpret the narratives of patients from minority groups, particularly when cultural differences shape expressions of distress [18]. While language analysis is routine in clinical psychiatry, producing reliable scientific data, generating fine-grained annotations, and conducting such analyses on large populations remain challenging.

Moreover, thematic analysis is particularly relevant in psychiatry because it resonates with the clinical focus on how people construct meaning from their experiences. As a qualitative method, thematic analysis identifies recurrent patterns in textual data and offers a structured approach to interpreting narratives through iterative annotation and inductive refinement of higher-order themes [19]. It can reveal phenomenological nuances during psychiatric disorders: how individuals experience emotions, self, embodiment, and social relatedness. The method has been used across diverse populations and data sources. However, when conducted manually, it is labor-intensive, often requiring trained coders or panels of expert clinicians to produce clinically validated themes [14]. It is also sensitive to clinician bias and typically constrained to small, monolingual corpora, limiting its scalability and generalizability [19].

Computational approaches to thematic analysis (commonly named topic modeling in natural language processing) represent documents in a shared vector space and discover structure via clustering or probabilistic topic models. Canonical methods (eg, latent Dirichlet allocation) treat documents as mixtures of latent topics [20]. Neural variants such as BERTopic embed textual data with transformer-based language models, reduce dimensionality, cluster, and summarize each cluster with keywords [21]. More recently, prompt-based frameworks delegate labeling to large language models (LLMs), yielding more specific, interpretable topic descriptions [22,23]. In mental health, topic modeling has been applied to peer-support forums [24], pandemic-era shifts in Reddit help-seeking [25], depression forums [26], and question-and-answer platforms [27]. Most relevant to our setting, it has been applied to free-response clinical speech, identifying depression-related topics in smartphone-collected recordings transcribed by automatic speech recognition [28].

Computational approaches offer 3 gains: reproducible annotation that limits researcher bias, substantial time savings over labor-intensive manual annotation, and the capacity to analyze larger datasets. However, many automated analyses remain shallow, relying on surface-level lexical statistics (eg, keyword counts and part-of-speech frequencies) to describe clusters [29], which can miss context, pragmatics (eg, sarcasm), and cultural idioms. Prior studies have also rarely addressed potential confounding factors. Language use may vary with demographics such as age and gender, independently of mental health status [30-32]. For example, adolescents with depression tend to discuss friends and school, whereas adults with depression might focus more on work and financial stressors [33]. Most topic modeling studies for mental health focus on a single language and data source (eg, one social media or one country’s clinical sample) [29,34-36]. Findings from a single cohort may not translate to other populations [37]. As a result, it remains unclear whether the topics identified in one group (eg, English-speaking social media users) also appear in other cultural or linguistic groups.

Fine-grained descriptions of clusters (eg, examination-related rumination and job-loss-related hopelessness) capture clinically relevant distinctions that broad, keyword-based descriptions (eg, sleep, work, and family) collapse together. We generated cluster descriptions using language models as they provide natural-language, human-readable labels, overcoming the interpretability limits of keyword-based topic models while retaining scalability for large corpora.

We developed a multilingual pipeline that (1) clusters spontaneous speech transcripts from 4 cohorts (general population and 3 clinical samples in French, Italian, Chinese, and Spanish), (2) generates fine-grained natural-language descriptions for each cluster, and (3) tests cluster membership for association with clinical scores and sociodemographic factors. We then quantified which questions best separate clinically relevant clusters, and we measured confounding by age, education, and sex, an aspect often overlooked in topic modeling despite strong demographic effects on language use.

Across these cohorts, cluster membership was associated with validated clinical scales, robustly in 3 cohorts and as an exploratory finding in the smaller Spanish cohort, and the language-model descriptions render the clusters readable.

## Methods

### Overview

In this study, we automatically clustered mental health transcripts from 4 distinct cohorts. Then, we generated descriptions of clusters to find fine-grained topics. Finally, we performed statistical analysis to find clinical differences between the clusters. Our automatic pipeline used language models for both clustering transcripts and generating cluster descriptions.

### Study Design

This is a secondary cross-sectional analysis of spontaneous speech transcripts from 4 independent observational cohorts: a large French general population sample (1809 assessments from 1338 participants) and 3 clinical populations collected in Italian (n=116), Chinese (n=52), and Spanish (n=90) languages. The data comprised transcription of participants’ spontaneous speech productions in response to open-ended questions and validated self- or clinician-administered psychiatric rating scales. In Table 1, we summarized statistics of demographics and clinical scores of the 4 cohorts. Categorical variables were compared with the Pearson chi-square test, and continuous variables were compared with the Kruskal-Wallis H test based on control and noncontrol groups.

Throughout, the unit of analysis for clustering is the individual transcript (1 participant may contribute several transcripts, 1 per interview question). In Table 1, the 3 clinical cohorts are summarized at the participant level (Italian n=116, Chinese n=52, and Spanish n=90), whereas the French general population is summarized at the assessment level (1809 assessments from 1338 participants), reflecting how each cohort was collected. The French demographic and clinical counts in the table are therefore per assessment. The number of transcripts analyzed per cohort and question, after removing duplicates and transcripts shorter than 20 characters, is reported in Multimedia Appendix 1, which also reports the participant, recording, and transcript counts for each cohort.

**Table 1.** Demographics and clinical scores of the 4 cohorts. Significance indicators test whether each variable differs between clinical (depressed/suicidal) and control groups within each cohort: *P*<.001 indicates a significant difference. For clinical scores (PHQ-9^a^), *P*<.001 reflects the expected clinical difference between groups. In the Chinese cohort, a PHQ-9 score above 10 was part of the patient inclusion criteria, so this difference partly reflects that inclusion criterion. Depression diagnosis is a clinician-assigned clinical diagnosis (see Methods for the criteria used in each cohort). A variable that defines a cohort’s clinical grouping, the PHQ-9 in the French cohort, the depression diagnosis in the Italian and Chinese cohorts, and the C-SSRS^b^ class in the Spanish cohort, is not tested against that grouping. Tests: Pearson chi-square (categorical) and Kruskal-Wallis H (continuous).

	General population (n=1809 assessments)	Androids (n=116)	MODMA^c^ (n=52)	VOCES^d^ (n=90)
Demographics				
Language	French	Italian	Chinese	Spanish
Age	*P*<.001	n.s.^e^	n.s.	*P*<.001
Mean (SD)	37.8 (18.2)	37.4 (12.0)	31.3 (9.2)	38.6 (14.9)
Range	18‐91	19‐71	18‐52	21‐76
Sex, n (**%**)	n.s.	n.s.	n.s.	n.s.
Female	1187 (66.2)	84 (72.4)	16 (31)	39 (43)
Male	595 (33.2)	32 (27.6)	36 (69)	48 (53)
Other	11 (0.6)	0 (0.0)	0 (0)	3 (3)
Education, n (**%**)	n.s.	n.s.	n.s.	N/A^f^
No diploma	52 (2.9)	11 (9.5)	7 (13)	N/A
Secondary	291 (16.2)	37 (31.9)	8 (15)	N/A
Higher short	213 (11.9)	52 (44.8)	0 (0)	N/A
Higher long	1236 (69.0)	16 (13.8)	37 (71)	N/A
Clinical evaluation				
C-SSRS	N/A	N/A	N/A	N/A
Suicidal risk, n (%)	N/A	N/A	N/A	60 (67)
No suicidal risk, n (%)	N/A	N/A	N/A	30 (33)
Depression diagnosis	N/A	N/A	N/A	N/A
Depression, n (%)	N/A	64 (55.2)	23 (44)	N/A
No depression, n (%)	N/A	52 (44.8)	29 (56)	N/A
PHQ-9	N/A	N/A	*P*<.001	*P*<.001
Mean (SD)	5.2 (4.6)	N/A	9.4 (8.5)	10.5 (6.8)
Range	0‐27	N/A	0‐25	0‐26
≥10, n (%)	290 (16.0)	N/A	N/A	N/A

a PHQ-9: Patient Health Questionnaire-9.

b C-SSRS: Columbia Suicide Severity Rating Scale.

c MODMA: multi-modal open dataset for mental-disorder analysis.

d VOCES: Spanish clinical cohort for suicide-risk research.

e n.s.: indicates *P*≥.05 (no significant difference between groups).

f N/A: not applicable.

### Recruitment

#### French General Population Cohort

The French general population cohort contained 1809 assessment visits from 1338 French-speaking participants (1161 contributed a single visit and the remainder 2 to 6 visits, with 1 participant assessed 16 times). The unit of analysis is the per-question transcript. As some participants contributed repeated visits, a one-visit-per-participant sensitivity analysis (Multimedia Appendix 2) leaves the associations between cluster membership and clinical scores essentially unchanged. They responded to open-ended questions, including the following: “describe your last 24 hours,” “describe a negative event that happened to you in the past,” “describe a positive event that happened to you in the past,” “describe a negative event or situation you think might happen in the future (wk, mo, or y),” “describe a positive event or situation you think might happen in the future (wk, mo, or y),” and “describe how you are feeling at the moment and how your sleep has been lately.”

Self-administered questionnaires assessed participants’ mental health status, including depressive symptoms using the Patient Health Questionnaire-9 (PHQ-9) [38] and the Beck Depression Inventory (BDI) [39]; anxiety using the Generalized Anxiety Disorder 7-item scale (GAD-7); insomnia using the Athens Insomnia Scale (AIS) [40]; and fatigue using the Multidimensional Fatigue Inventory (MFI) [41]. Using the PHQ-9 score, we obtained a control group (PHQ-9 ≤9) and a probable-depression group (PHQ-9 ≥10). PHQ-9 was at or above 10, the conventional threshold for probable depression, in 290 of 1809 (16%; 221 of 1338, 16.5% participants at first visit) assessments.

Participants completed a series of speech tasks and self-administered questionnaires through a mobile research app specifically designed for clinical studies.

#### Italian Clinical Population (Androids) Cohort

The Androids cohort was specifically designed for automatic depression detection from speech [42], and includes 228 recordings from 116 native Italian speakers, collected across 2 tasks (a spontaneous interview task and a read-speech task). We analyzed the spontaneous interview task only. After removing duplicates and transcripts shorter than 20 characters, 110 transcripts entered the clustering analysis (Multimedia Appendix 1). Of the 116 participants, 64 participants were clinically diagnosed with depression by professional psychiatrists, providing a reliable characterization of individuals with depression compared to self-reported assessments. The interview task involved answering questions about everyday life (eg, “What did you do last weekend?”).

Participants were native Italian speakers recruited across 5 Mental Health Centers in Italy. Depression was diagnosed by psychiatrists according to *DSM-5* (*Diagnostic and Statistical Manual of Mental Disorders* [Fifth Edition]) criteria, spanning major depressive disorder (MDD), bipolar disorder in a depressive phase, and related depressive conditions [42]. The Androids corpus provides this binary clinical diagnosis (depressed vs control). Controls reported no prior mental health history and were selected to match the patient group on age, gender, and education.

#### Chinese Clinical Population (MODMA) Cohort

The multi-modal open dataset for mental-disorder analysis (MODMA) cohort [43] included 52 Chinese-speaking participants, with 23 outpatients diagnosed with MDD (16 males and 7 females, aged 18‐52 years) and 29 healthy controls (20 males and 9 females, aged 19‐52 years). MDD diagnoses were confirmed by clinical psychiatrists at Lanzhou University Second Hospital based on the Mini-International Neuropsychiatric Interview [44] and *DSM-IV* (*Diagnostic and Statistical Manual of Mental Disorders* [Fourth Edition]) diagnostic criteria [45].

**Figure 1.**
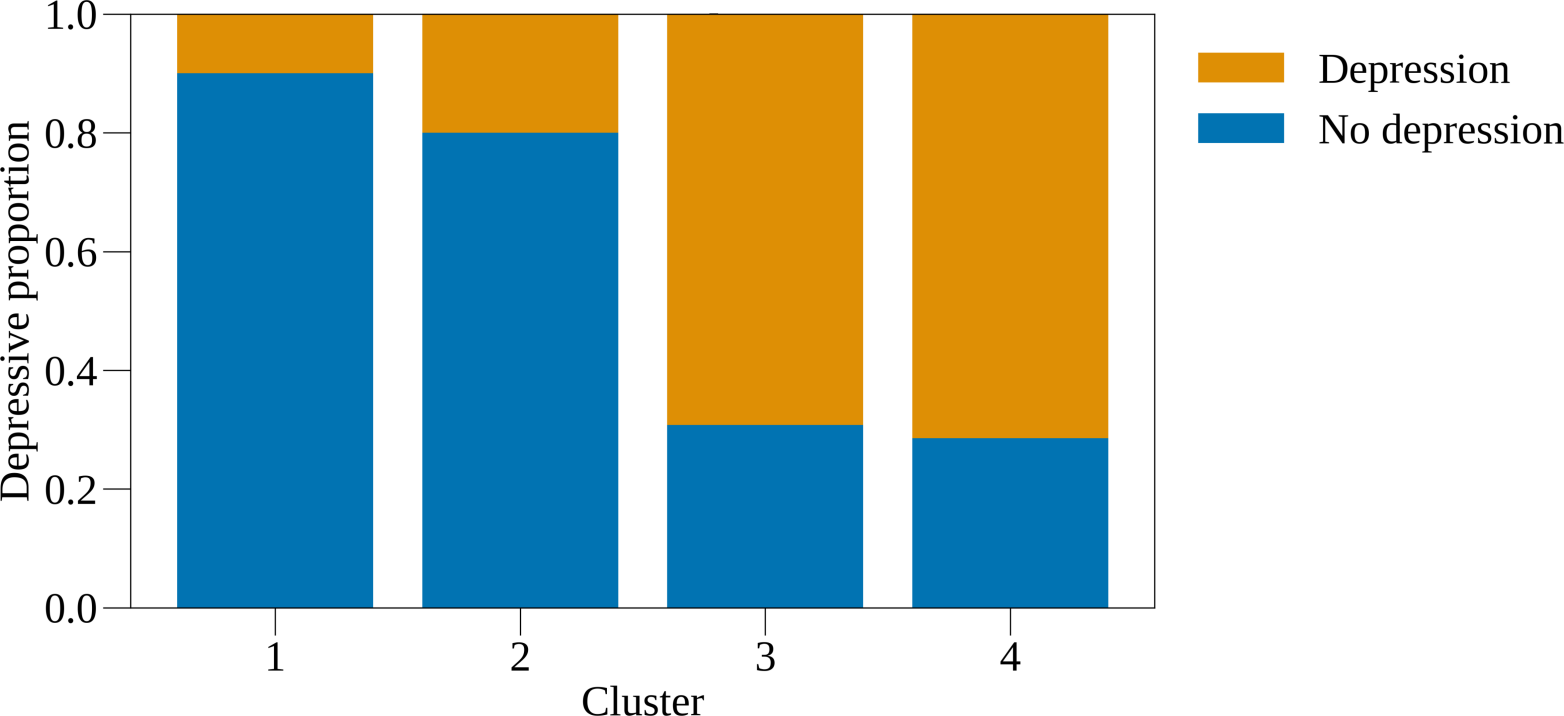
Proportion of participants with depression across automatically identified clusters in the MODMA cohort (n=52 transcripts, Chinese clinical population). Responses to the neutral-valence interview questions (each participant’s answers to that valence group concatenated; see Methods section). Depression status is a clinician-assigned MDD diagnosis (MINI/*DSM-IV* criteria). Effect size Cramér V=0.55 (95% CI 0.36 to 0.78, *P*=.001). Cluster descriptions generated by Qwen3-14B summarizing 30 random transcripts per cluster. In cluster 1 (age 26±6 years, n=10), the individuals express a range of emotional states from mild anxiety and stress related to academic or work pressures to generally positive moods, emphasize the significance of maintaining healthy family relationships and having supportive friends or mentors, acknowledge personal traits such as being easily irritable, argumentative, or overly accommodating, and outline specific plans involving career development, returning to hometowns, or improving personal skills such as research capabilities or education. In cluster 4 (age 28±6 years, n=14), the individuals express how physical health issues and emotional struggles such as depression, anxiety, and loss of motivation significantly impact their daily lives, relationships, and work, while also highlighting the complex role of supportive friends and family, as well as persistent feelings of inadequacy and communication challenges within familial contexts. *DSM-IV*: *Diagnostic and Statistical Manual of Mental Disorders* (Fourth Edition); MDD: major depressive disorder; MINI: Mini-International Neuropsychiatric Interview; MODMA: multimodal open dataset for mental-disorder analysis.

Speech samples consisted of responses to 18 interview questions derived from *DSM-IV* and the Hamilton Depression Rating Scale [46]. Following the dataset design [43], these questions are grouped by emotional valence into 6 positive, 6 neutral, and 6 negative questions. The analysis reported here and shown in Figure 1 uses the neutral-valence questions, everyday topics such as “describe one of your friends, including age, job, character, and hobbies.” For each participant, the answers to the questions within a valence group were concatenated into a single transcript before clustering. The positive-valence questions (such as “What is the best gift you have ever received, and how did you feel?”) and the negative-valence questions (such as “What makes you desperate?”) were analyzed separately (Multimedia Appendix 3).

Inclusion criteria included a PHQ-9 score above 10 and no psychotropic drug treatment in the 2 weeks before participation. Healthy controls were recruited through public advertisements, with exclusion criteria that ruled out personal or family histories of mental disorders.

#### Spanish Clinical Population (VOCES) Cohort

The Spanish clinical cohort for suicide-risk research (VOCES) cohort [47] comprised 90 Spanish-speaking adults who responded to open-ended questions such as: “Can you recall some recent good news you had and how did that make you feel?” “Do you get a characteristic feeling when you’re sad or down, and what do you normally do to cheer yourself up?” and “Describe your average day.”

**Figure 2.**
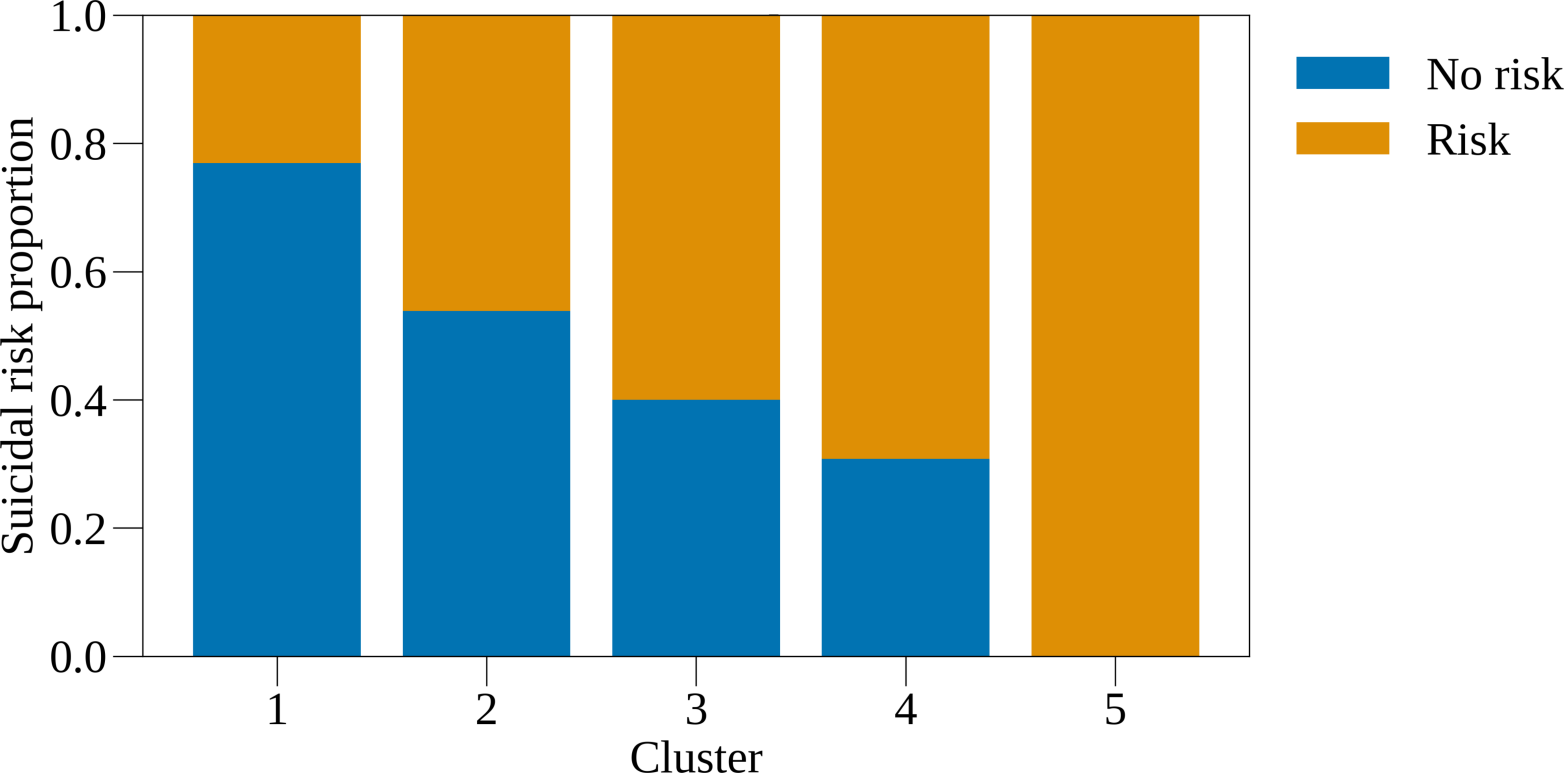
Proportion of participants with suicidal risk across automatically identified clusters in the VOCES cohort (n=58 transcripts, Spanish clinical population). Responses to “do you get a characteristic feeling when you’re sad or down, and what do you normally do to cheer yourself up?” Suicidal-risk class (any positive C-SSRS finding vs none), assessed by psychiatrists with the Spanish C-SSRS. Due to the small sample, this association is unstable across clustering seeds, so it is treated as exploratory and its effect size is the seed-averaged value (mean Cramér V=0.27; Multimedia Appendix 4). The figure shows the primary clustering, the single run on which the cluster descriptions and the plotted proportions are based, and for this run Cramér V=0.50 (95% CI 0.37 to 0.71, *P*=.006). Cluster descriptions generated by Qwen3-14B summarizing 30 random transcripts per cluster. In cluster 1 (age 32±13 years, n=13), the individuals express common strategies for coping with sadness or low mood, including mindfulness practices (meditation or breathing exercises), seeking social support (talking to friends or loved ones), engaging in physical activity or rest (sleep or exercise), and allowing emotions to pass naturally without forced suppression or avoidance. In cluster 5 (age 34±11 years, n=9), the individuals express common strategies for coping with low moods, including using media (eg, television, YouTube, and music), engaging in physical activities (eg, walking or cleaning), seeking social support (eg, talking to friends or family), and using distraction techniques (eg, gaming or napping) to avoid rumination, while some emphasize the importance of acceptance, structured routines, and medication in managing emotional distress. C-SSRS: Columbia Suicide Severity Rating Scale; VOCES: Spanish clinical cohort for suicide-risk research.

Suicidal risk was assessed by professional psychiatrists using the validated Spanish version of the Columbia Suicide Severity Rating Scale (C-SSRS) [48,49]. Each participant’s summed C-SSRS severity score ranged from 0 to 21 in this cohort (mean 9.2, SD 7.5). For analysis, participants were dichotomized into a suicidal-risk group, defined as any positive C-SSRS finding (a score of 1 or more, indicating suicidal ideation or behavior), and a no-risk group (a score of 0). This binary risk class is the outcome tested in Figure 2. A total of 60 of the 90 (67%) participants were in the suicidal-risk group.

Inclusion criteria required participants to be at least 18 years of age, fluent in Spanish, and capable of understanding and signing the informed-consent form. Psychiatric outpatients were recruited consecutively during routine consultations, and healthy volunteers were enrolled through snowball sampling among mental-health staff and university students.

### Analysis

#### Overview

**Figure 3.**
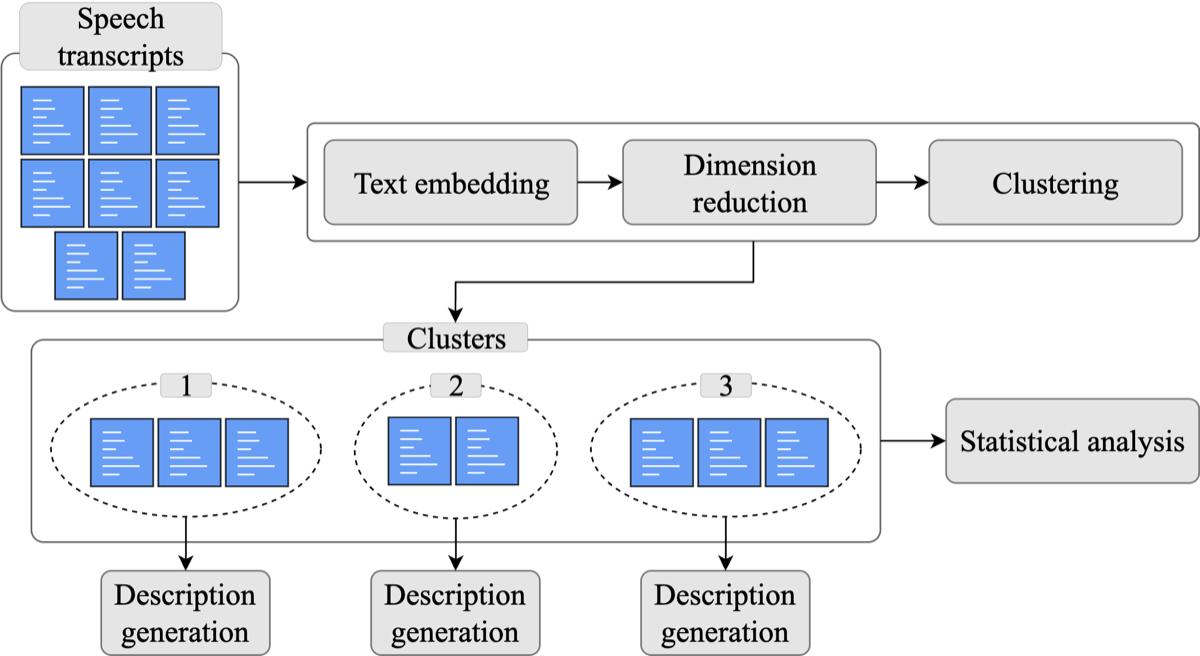
Computational pipeline for semantic clustering and description generation. Speech transcripts are converted to semantic vectors via multilingual embeddings, dimensionally reduced, and grouped into clusters using density-based methods. Each cluster undergoes: (1) statistical analysis linking cluster membership to clinical and demographic variables, and (2) automatic description generation where a large language model summarizes transcripts per cluster into human-readable natural language. This dual quantitative-qualitative output enables both statistical hypothesis testing and human-readable interpretation of discovered topics (Figures 1, 2, 4, and 5).

We adopted a data-driven pipeline that clusters transcripts based on their semantic similarity using multilingual language models (Figure 3). No machine translation was performed as transcripts were analyzed in the original language. Hyperparameters for each step are available in Multimedia Appendix 5.

**Figure 4.**
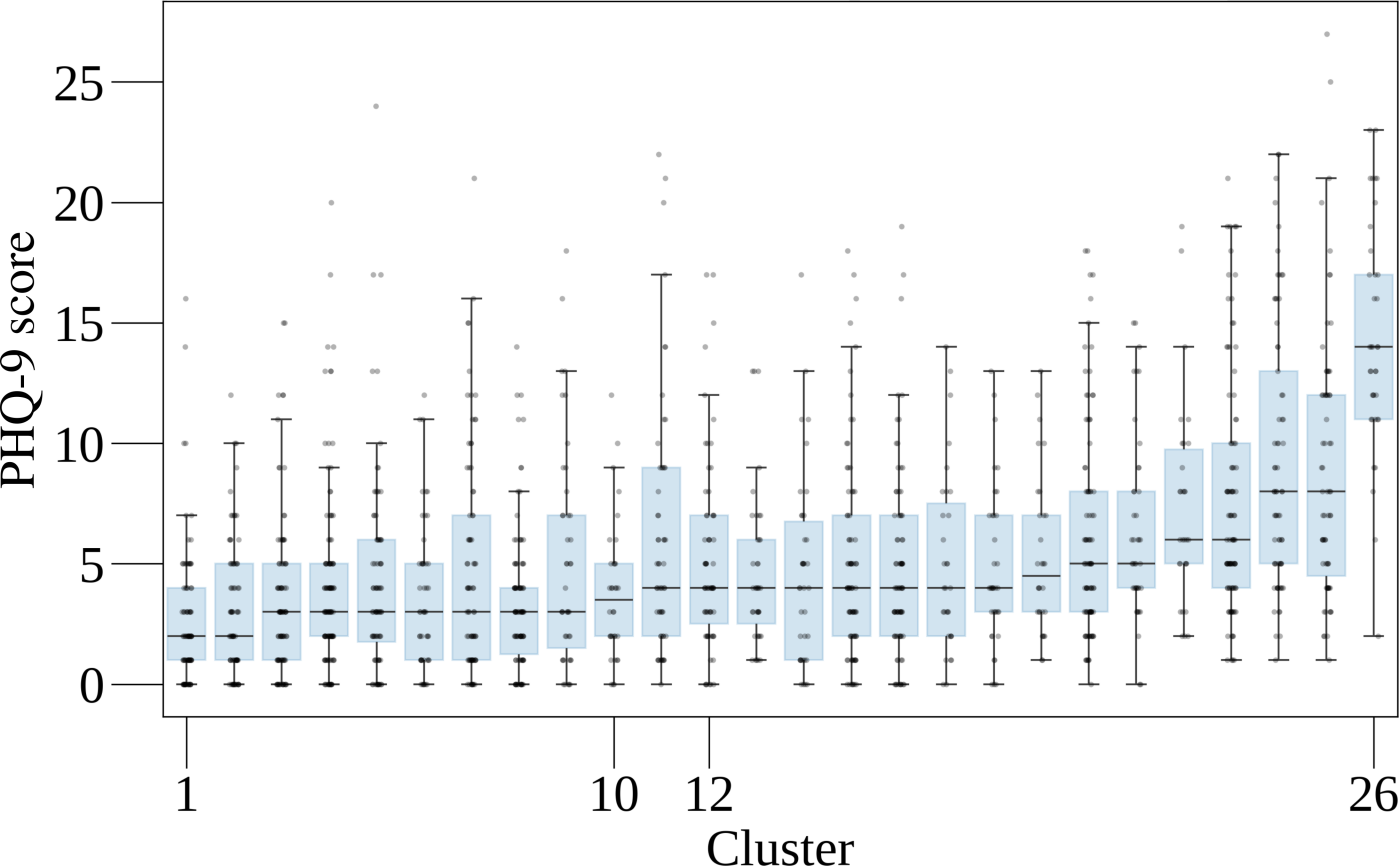
Distribution of PHQ-9 depression scores across automatically identified clusters in the French general population cohort (n=1645 transcripts in 26 clusters, after excluding HDBSCAN noise). Responses to the prompt “describe how you are feeling at the moment and how your sleep has been lately.” Box plots show median (center line), IQR (box), and 1.5× IQR whiskers. Effect size η²=0.19 (95% CI 0.17 to 0.24, *P*<.001) indicates a large difference between clusters per Cohen conventions. Sample sizes per cluster range from 34 to 92. Selected cluster descriptions (clusters 1, 10, 12, and 26) were generated by Qwen3-14B summarizing 30 random transcripts per cluster. In cluster 1 (age 39±19 years, n=92), the individuals express consistent satisfaction with their current well-being, emphasizing good sleep quality, restful or pleasant nights, and a general sense of relaxation, even when noting variations in sleep duration or occasional fatigue. In cluster 10 (age 69±15 years, n=34), the individuals express frequent nighttime urinary interruptions disrupting sleep, often attributed to age-related conditions such as prostate issues or overactive bladder, alongside mixed reports of physical well-being, mental resilience, and lifestyle factors such as retirement or exercise influencing their overall health and sleep patterns. In cluster 12 (age 24±9 years, n=67), the individuals express stress related to academic examinations, significant life decisions, and workloads, alongside sleep disturbances caused by lifestyle changes, increased responsibilities, or environmental adjustments, while some also highlight temporary relief from pressures through personal achievements or upcoming positive events. Cluster 26 (age 25±9 years, n=37), the individuals express sleep disturbances characterized by insomnia, frequent awakenings, and restless sleep, alongside pervasive anxiety, emotional instability, and self-esteem issues, which collectively contribute to persistent fatigue, impaired daily functioning, and a diminished sense of well-being. HDBSCAN: Hierarchical Density-Based Spatial Clustering of Applications With Noise; PHQ-9: Patient Health Questionnaire-9.

**Figure 5.**
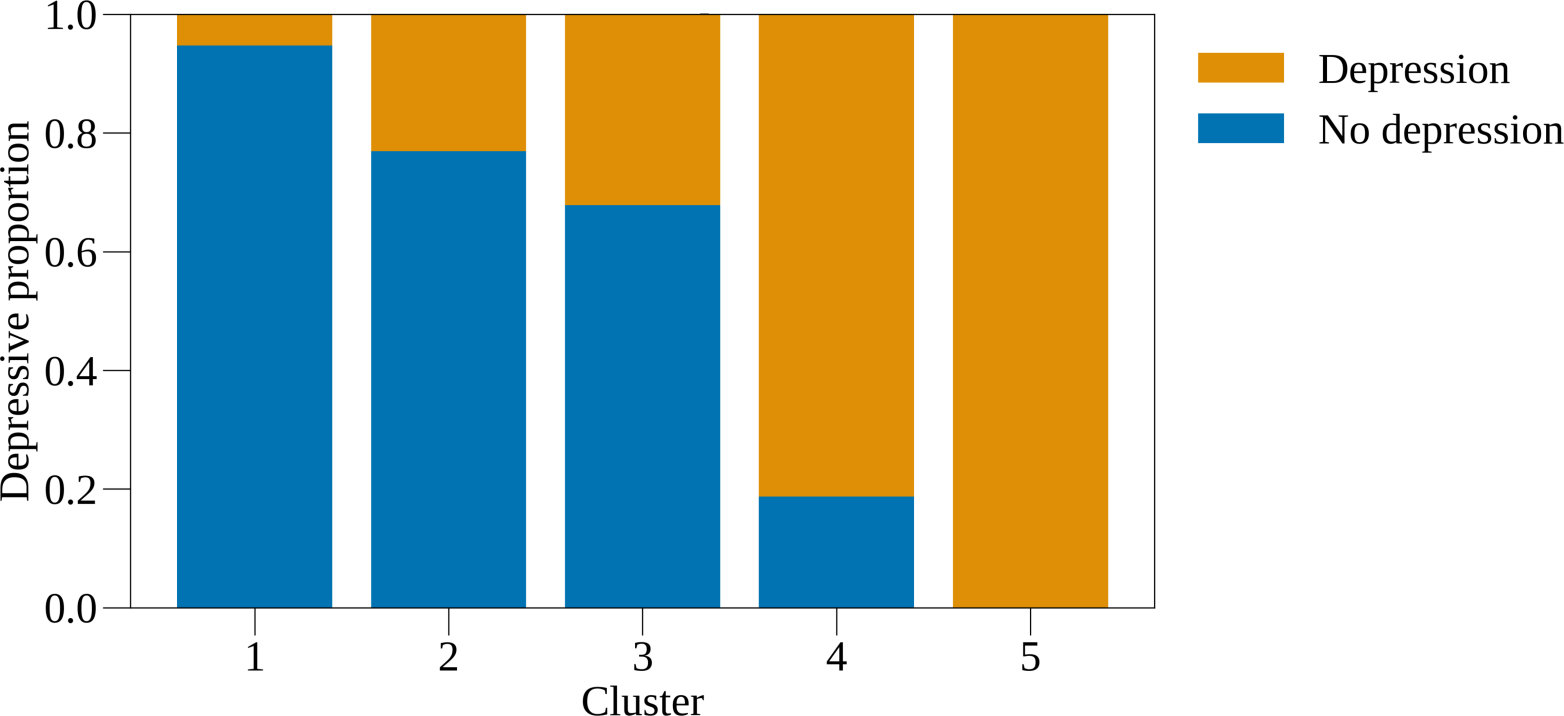
Proportion of participants with depression across automatically identified clusters in the Androids cohort (n=106 transcripts, Italian clinical population, after excluding HDBSCAN noise). Responses to questions about everyday life (eg, “What did you do last weekend?”). Depression status is a clinician-assigned *DSM-5* diagnosis. Effect size Cramér V=0.74 (95% CI 0.65 to 0.85, *P*<.001). Cluster descriptions generated by Qwen3-14B summarizing 30 random transcripts per cluster. In cluster 1 (age 44±11 years, n=19), the individuals express a shared focus on family dynamics and intergenerational relationships, regional identity and emotional ties to Naples, and the pursuit of personal passions such as music, research, and education, while reflecting on the complexities of balancing domestic responsibilities with professional or creative endeavors. In cluster 5 (age 50±11 years, n=30), the individuals express persistent struggles with depression, anxiety, and panic attacks, often exacerbated by significant life events such as family responsibilities, health-related stress from routine medical check-ups, and a pervasive sense of stagnation and purposelessness amid repetitive daily routines, while some also grapple with disrupted academic or career goals and complex family dynamics. *DSM-5*: *Diagnostic and Statistical Manual of Mental Disorders* (Fifth Edition); HDBSCAN: Hierarchical Density-Based Spatial Clustering of Applications With Noise.

#### Audio Transcription

We transcribed all speech recordings with Whisper large-v3-turbo, a state-of-the-art neural model for automatic speech recognition [50]. We removed duplicates and small transcripts (less than 20 characters).

#### Text Embedding

To obtain multilingual semantic representations, each transcript was mapped to a 4096-dimensional vector (also called an embedding) using Qwen3-Embedding-8B, a language model for text embedding [51]. We selected this model because, as of June 2025, it ranked first on the multilingual track of the Massive Text Embedding Benchmark [52], covering all 4 languages of our cohorts, and because it is released under a permissive open-weight (Apache-2.0) license, which allowed us to run the model ourselves, a requirement for processing sensitive clinical speech without transmitting data to any third-party or commercial service. The model turned every transcript into a point in a vector space: transcripts dealing with similar issues have similar vectors; hence, the clustering step can identify clusters that share the same topics.

#### Dimension Reduction

Dimension reduction transforms high-dimensional embeddings into a lower-dimensional representation that approximately preserves their geometric structure, thereby simplifying clustering and visualization. We projected the embeddings to a 5D space using UMAP (Uniform Manifold Approximation and Projection) [53]. Reducing embeddings with UMAP before density-based clustering is standard practice in modern topic modeling and is the default pipeline in BERTopic [21]. This step facilitates clustering, as we reduced the search space from 4096 to 5 dimensions. The number of components is not a sensitive design choice: the clinical associations are essentially unchanged when it is varied from 2 to 10. The choice of reduction method does matter. Replacing UMAP with a linear (principal component analysis) or alternative nonlinear (t-distributed stochastic neighbor embedding) reduction recovers weaker associations, because neither preserves the local neighborhood density that density-based clustering relies on (Multimedia Appendix 4). A neighborhood-preserving nonlinear reduction such as UMAP is therefore an important part of the method, whereas its exact dimensionality is not.

#### Clustering

Clustering consisted of finding groups of patients with semantically similar transcripts. These transcripts may contain common topics that are clinically relevant. We extracted clusters using HDBSCAN (Hierarchical Density-Based Spatial Clustering of Applications With Noise) [54], an algorithm that (1) requires no a priori specification of the number of clusters, (2) adapts to variable-density clusters, and (3) labels ambiguous points as noise.

#### Cluster Description

We automatically labeled each cluster by randomly sampling 30 transcripts and asking a language model to summarize them, focusing on what is common between the transcripts. This sample size was chosen because (1) descriptions generated from 30 transcripts are reproducible across resampling, repeated generation, and prompt wording (Multimedia Appendix 6), and (2) it approaches the maximum input that fits within the model’s context window (40,960 tokens for Qwen3-14B). As clustered transcripts are semantically similar by construction, 30 samples typically capture the cluster’s core themes without redundancy. The model received only the transcripts, so the generated descriptions are produced independently of any clinical score or demographic variable. As with the embedding model, we required an open-weight model under a permissive license, so that sensitive clinical speech was never sent to a third-party or commercial service. We used Qwen3-14B [55], an instruction-tuned 14-billion-parameter model from a leading open-weight family, with strong multilingual coverage and a context window large enough to take the 30-transcript input in a single pass. We automatically obtained textual descriptions of clusters that contain several topics. This generative approach yields fine-grained topic descriptions automatically and at scale, going beyond keyword-based descriptions, which stay coarse, and manual coding, which does not scale. The prompt is provided in Multimedia Appendix 5.

#### Qualitative Description

A board-certified psychiatrist (one of the coauthors) read the generated cluster descriptions as a coherence check, a qualitative reading rather than a clinical validation.

#### Statistical Analysis

Each transcript is associated with a clinical score or diagnosis, such as a PHQ-9 score or a clinician-assigned depression diagnosis. Each cluster is therefore associated with a distribution of clinical scores. We performed statistical tests to determine whether there are statistical differences between the clusters based on their clinical scores. Transcripts that HDBSCAN labeled as noise were excluded from these cluster-level tests.

For continuous scores such as PHQ-9, we performed a Kruskal-Wallis H test to determine whether the clusters follow the same distribution or not. If we rejected the null hypothesis, we performed a pairwise Dunn test (with Benjamini-Hochberg corrections) to find significant differences between clusters. We reported effect sizes using eta-squared (η²=(H−k+1)/(n−k), where H is the Kruskal-Wallis statistic, k the number of clusters, and n the number of transcripts), interpreted following Cohen conventions: 0.01=small, 0.06=medium, and 0.14=large [56]. We also report adjusted *P* values.

For categorical outcomes such as a clinician-assigned depression diagnosis, we tested whether the distribution of the outcome differed across clusters using a chi-square test of independence. If the omnibus test was significant, we performed pairwise chi-square tests with Benjamini-Hochberg corrections. We report effect sizes using Cramér V and adjusted *P* values. For the small clinical cohorts, where sparse expected cell counts can violate the chi-square large-sample assumption, we also report the expected counts and confirm each principal association with an assumption-free Monte-Carlo permutation chi-square (Multimedia Appendix 7).

We applied 2 Benjamini-Hochberg false-discovery-rate corrections. Within each significant omnibus test, the pairwise comparisons (pairwise Dunn or pairwise chi-square) were corrected together as one family. Across the question-by-clinical-score grid in Multimedia Appendix 3, the omnibus *P* values were corrected jointly across all cells within a cohort. These omnibus tests and effect sizes are exploratory rather than prespecified: this study was designed to identify which questions and clinical constructs yield clusters associated with clinical status, not to test a small set of hypotheses fixed in advance.

We report effect sizes from the primary clustering, the single pipeline run on which all figures and cluster descriptions are based. Where reclustering is unstable, as for the Spanish suicide-risk association, we instead report the seed-averaged value and label the finding exploratory. Multimedia Appendix 4 shows these associations are robust to the clustering seed and reports a bootstrap 95% CI for every effect size stated in this paper. These CIs are conditional on the reported clustering: they quantify the sampling uncertainty of an effect size for a fixed partition and do not capture the additional uncertainty from rerunning the clustering or from choosing which interview questions to report. Uncertainty from reclustering is characterized separately by the seed-stability analysis (Multimedia Appendix 4), and the choice of questions and scores is exploratory rather than prespecified. The CIs are therefore widest, and read most cautiously, in the small clinical cohorts. Throughout the Results section, a parenthetical range accompanying an effect size is its 95% CI (for example, η²=0.19, 0.17 to 0.24).

### Ethical Considerations

#### Overview

All 4 cohorts used in this work had previously obtained informed consent from participants and ethical approval for speech data collection and secondary analysis.

#### Data Governance

All computational processing used open-weight models (Whisper large-v3-turbo, Qwen3-Embedding-8B, and Qwen3-14B) that the authors ran themselves on controlled computing infrastructure. No transcripts, audio recordings, or derived data were transmitted to any third-party or external commercial API service at any stage of the pipeline. Data were stored without directly identifying information.

#### French General Population Cohort

All participants provided informed consent in accordance with the Declaration of Helsinki, Good Clinical Practice guidelines, and local regulations. This study received approval from the French National Institutional Review Board (identifier 23.00748.OOO2L7#I). Data was securely stored without identifying information, and participants received a gift card for their time.

#### Italian Clinical Population (Androids) Cohort

All participants volunteered and provided written informed consent in accordance with Italian and European Union data-protection law. The protocol was approved by the Ethics Committee of the Department of Psychology, Università degli Studi della Campania “Luigi Vanvitelli” (protocol 09/2016).

#### Chinese Clinical Population (MODMA) Cohort

All recordings were collected under ethical guidelines approved by the Ethics Committee of the Second Affiliated Hospital of Lanzhou University. Participants provided written informed consent and received approximately US $16 as compensation.

#### Spanish Clinical Population (VOCES) Cohort

All procedures were approved by the Ethics Committee of the University Hospital Fundación Jiménez Díaz (PIC128-21_FJD) and complied with the Declaration of Helsinki. Participants provided written informed consent, and this study involved no costs or monetary compensation.

## Results

### Overview

We present results at 3 complementary levels: (1) quantitative identification of clusters with statistically significant differences in clinical scores and demographics, (2) automatically generated natural-language descriptions of cluster content (presented alongside quantitative results), and (3) a qualitative reading of the descriptions by a board-certified psychiatrist among the coauthors (qualitative description of clusters).

### Some Clusters Are Associated With High and Low Clinical Scores

Each transcript is associated with clinical scores. Each cluster of transcripts is thus a distribution of scores that can be compared to other distributions. Across all 4 cohorts, unsupervised clustering of transcript embeddings yielded between 4 and 26 distinct semantic clusters per cohort-question combination.

Cluster membership was significantly associated with clinical outcomes in the French, Italian, and Chinese cohorts, separating clusters with high and low clinical scores (Figures 1, 4, and 5). In the smaller Spanish cohort, the association was unstable across clustering seeds and is reported as exploratory, by its seed-averaged effect size (mean Cramér V=0.27; Figure 2).

For the French general population cohort based on answers to “Describe how you are feeling at the moment and how your sleep has been lately,” among 26 identified clusters (n=1645 nonnoise transcripts), PHQ-9 scores differed significantly across clusters (η²=0.19, 0.17 to 0.24, *P*<.001). Cluster 26 (n=37, age 25±9 years) exhibited the highest depression scores (PHQ-9 13.4±5.4) with LLM-generated descriptions highlighting sleep disturbances characterized by insomnia, frequent awakenings, and restless sleep, alongside pervasive anxiety, emotional instability, and self-esteem issues. In contrast, cluster 1 (n=92, age 39±19 years) showed the lowest depression scores (PHQ-9 2.6±2.2), with descriptions emphasizing consistent satisfaction with current well-being, good sleep quality, and general relaxation.

Intermediate-scoring clusters showed age-specific thematic content: cluster 10 (PHQ-9 5.8±4.3, age 69±15 years, n=34) contained descriptions of frequent nighttime urinary interruptions attributed to age-related conditions, while cluster 12 (PHQ-9 6.2±4.1, age 24±9 years, n=67) described stress related to academic examinations and significant life decisions.

We also report clusters with the highest and lowest clinical scores along with their generated descriptions for the clinical populations: Androids (Figure 5), MODMA (Figure 1), and VOCES (Figure 2).

### Some Questions Are Better Suited to Find Clinical Differences

We calculated effect sizes across questions and clinical scores (eg, sociodemographics such as age and education) for each cohort, with a short observation under each heatmap (Multimedia Appendix 3). We found that some questions are better suited to finding significant differences between clusters.

For example, for the French general population, the question “Describe how you are feeling at the moment and how your sleep has been lately” is the one with the highest effect sizes (eta-squared, η²) across clinical scores such as AIS (0.15, 0.13 to 0.20), BDI (0.16, 0.14 to 0.21), GAD-7 (0.16, 0.14 to 0.21), MFI (0.14, 0.12 to 0.19), and PHQ-9 (0.19, 0.17 to 0.24; Multimedia Appendix 3). Every one of these associations was significant at *P*<.001 and remained so after the Benjamini-Hochberg false-discovery-rate control across the effect-size grid (Multimedia Appendix 3), consistent across clinical scores. The method captures how participants describe their sleep and mood, not just whether they report problems. For instance, clusters distinguished age-related nocturia from anxiety-driven insomnia, distinctions impossible with binary screening questions. We showed the distribution of the PHQ-9 score for this question (Figure 4).

In contrast, questions about past or future events yielded small eta-squared effect sizes. For example, topics extracted from “describe a negative event that happened to you in the past” showed: AIS (η²=0.01, 0.01 to 0.04), BDI (η²=0.01, 0.01 to 0.05), GAD-7 (η²=0.02, 0.02 to 0.06), MFI (η²=0.01, 0.01 to 0.05), and PHQ-9 (η²=0.03, 0.02 to 0.06). Similarly, questions about positive or negative future events did not produce clinically significant clusters. This question-selection pattern is robust to the clustering seed: across 10 random seeds, only the feelings-and-sleep question retains a substantial PHQ-9 association (mean η²=0.20), while the past- and future-event questions remain at or below η²=0.05 (Multimedia Appendix 4).

### Age, Education, and Sex Are Systematically Associated With Topic Content Independently of Clinical Status

Beyond clinical scores, sociodemographic factors were also significantly associated with cluster membership.

This analysis was possible only in the French general population, the only cohort large enough to stratify participants across all subgroups and diagnoses. We examined whether sociodemographic factors (age, education, and sex) confound the association between cluster membership and clinical scores. Cluster 26 (highest PHQ-9; Figure 4) consisted predominantly of young adults (age 25±9 years), so we tested directly whether this cluster reflects depression-specific language or age-typical stressors within a population with depression.

To disentangle these effects directly, we fitted, for each clinical score, ordinary least-squares models of the score on cluster membership, on demographics (age, sex, and education), and on both, with SEs clustered by participant to account for repeated visits (Multimedia Appendix 2). Cluster membership remained strongly associated with every score after adjustment and explained more variance than demographics: for PHQ-9 it accounted for 23.2% of the variance against 5.3% for demographics, and its partial η² changed little after adjustment (0.232 unadjusted to 0.220 adjusted, 0.188 to 0.279; joint test of cluster membership *F*=12.9, *P*<.001). The same pattern held for all other scores and in a one-visit-per-participant sensitivity analysis. The demographic contribution is therefore genuine but partial, not one that accounts for the association between cluster membership and clinical scores entirely.

We also found that all questions led to significant differences between clusters for sociodemographics such as age, education, and sex (Multimedia Appendix 3). There are some exceptions: “describe a negative event that happened to you in the past” did not have a significant difference for education, and “describe a positive event that happened to you in the past” did not have a significant difference for sex.

The question “describe your last 24 hours” had the highest effect size for age (η²=0.27, 0.24 to 0.32) and sex (Cramér V=0.22, 0.21 to 0.30). The following description corresponded to the cluster with the lowest mean age, mostly comprising young participants (age 22±5 years, n=37):

The individuals express involvement in professional or academic internships, collaboration with colleagues or mentors, and routine-based daily activities such as work tasks, social interactions, and personal responsibilities.

The following description corresponded to the cluster with the highest mean age, mostly comprising middle-aged and senior participants (age 55±15 years, n=56):

The individuals express common topics such as engaging in routine household tasks such as cooking, cleaning, and laundry, participating in family-centered activities such as shared meals and playing games with children, and allocating time to leisure pursuits, including watching television, movies, or using electronic devices for entertainment.

In both examples above, the cluster descriptions contain age-related topics.

### Qualitative Description of Clusters

The cluster descriptions are an interpretability layer, generated from the transcripts alone using a clinically agnostic prompt that never sees clinical scores or diagnoses. A board-certified psychiatrist (one of the coauthors) reviewed them and found them coherent with the clinical scores: across cohorts, clusters with higher scores carried descriptions of negative emotional states, functional impairment, and disrupted relationships, while lower-risk clusters emphasized positive coping strategies and supportive relationships. On the same clusters, the language-model descriptions state the clinical character of a cluster that keyword labels leave implicitly (Multimedia Appendix 8). We separately examined whether the descriptions are grounded in the transcripts they summarize, rather than asserting unsupported content. Of the 40 cluster descriptions, 39 were faithful to their transcripts, the single exception imposing an organizing structure its cluster does not have (Multimedia Appendix 9).

For example, in the Italian population (Figure 5), in the cluster with the highest proportion of depressive participants (cluster 5), the individuals expressed persistent struggles with psychiatric symptoms, exacerbated by significant life events, a sense of stagnation and purposelessness, and difficulties in professional and family functioning, whereas in the cluster with the lowest number of depressive participants (cluster 1), they highlighted the importance of family relationships, cultural affiliations, and personal interests.

## Discussion

### Principal Results

We presented a data-driven approach that discovers human-readable topic descriptions across general and clinical populations using language models. To our knowledge, this is the first study that automatically produces interpretable descriptions of clusters of spontaneous speech in different populations. We make 2 distinct claims. First, resting only on cluster membership (fixed by the embeddings and clustering of the transcripts), score-agnostic clusters of spontaneous speech are associated with clinical scores and separate validated scales in the French, Italian, and Chinese cohorts, with an exploratory association in the smaller Spanish cohort. Second, the language-model descriptions add interpretability, rendering those clusters in human-readable language more directly than keyword lists. Every statistical result belongs to the first claim and stands on its own, independent of how the clusters are described.

Three findings stand out. First, unsupervised clustering of transcript embeddings yields clusters that significantly associate with clinical scores in the French, Italian, and Chinese cohorts. Second, certain interview questions, for example “describe how you are feeling at the moment and how your sleep has been lately,” are more effective at yielding clusters associated with clinical status. Third, sociodemographic factors (age, education, and sex) systematically associate with cluster membership independently of clinical status, revealing that topic modeling captures both mental health phenomena and normative life circumstances, a distinction rarely examined in computational psychiatry. Beyond these, the pipeline automates the labor-intensive early stages of thematic analysis and surfaces, in a data-driven multilingual way, risk and protective topics consistent with those that resource-intensive expert consensus previously established by hand.

This third finding is central to interpreting the results. As age, sex, and education are themselves associated with cluster membership, a cluster associated with clinical status may be so in part through demographically patterned content. The clearest example is cluster 26 in the French cohort (highest PHQ-9), which is composed predominantly of young adults (age 25±9 years). Its “depression-associated” content may partly reflect age-typical stressors occurring in a depressed subgroup rather than depression-specific language per se. We therefore treat the demographic-confound analysis as a core result: a covariate-adjusted analysis (Results section and Multimedia Appendix 2) shows the association between cluster membership and clinical scores survives adjustment for age, sex, and education, and we return to its limits in the Limitations section.

### Content Not Captured by the Clinical Scales

Not every question or cluster was strongly associated with the clinical scales, and these weaker results are themselves informative. The questions about past or future events yielded stable clusters (Multimedia Appendix 4) whose descriptions summarize life circumstances, yet their associations with the symptom scores were small (η² at or below 0.05; Multimedia Appendix 3). Sociodemographic factors likewise shaped topic content independently of clinical status. In both cases, the pipeline recovered coherent content from speech that the symptom scales do not measure. We read this not as a shortcoming of the pipeline but as a reminder that a clinical scale quantifies a predefined set of symptoms and does not capture the full complexity of the lived experience of depression. Standard depression scales share little of their symptom content with one another [57]. What patients consider recovery extends beyond symptom relief [8]. First-person accounts of the lived experience of depression describe dimensions that no symptom scale measures [14]. As the descriptions render this content in human-readable language rather than reducing it to a score, the approach reports content that a purely score-based analysis would leave out.

### Limitations

#### Overview

We set out the boundaries of this study and how they shape its interpretation.

#### Sample Sizes and Representativeness

The clinical cohorts are modest in size (n=52‐116). We therefore tested cluster stability directly: a stability analysis across random seeds and minimum cluster sizes, covering every interview question in all 4 cohorts, together with internal cluster-validity indices for each cohort’s principal clustering, is reported in Multimedia Appendix 4. The French general population cohort, while larger (n=1809), over-represents highly educated participants. Larger-scale investigations with more balanced recruitment are needed to establish population-level generalizability.

#### Confound Analysis

We performed the covariate-adjusted confound analysis only on the French general population cohort, as the clinical cohorts lacked sufficient sample sizes for it. In the French cohort, the association between cluster membership and clinical scores survived adjustment for age, sex, and education (Results section and Multimedia Appendix 2). Whether the same holds in the clinical samples remains open, because their size did not support the adjustment there. Extending covariate-adjusted or age-matched designs to the clinical cohorts is the next step. Future work should also incorporate demographic covariates directly into clustering.

#### Heterogeneity of Clinical Measures

Our cohorts used different clinical instruments: PHQ-9 (self-reported depression severity), a clinician-assigned depression diagnosis, and the C-SSRS (suicide risk). These measure related but distinct constructs, limiting direct cross-cohort comparisons. This heterogeneity reflects the reality of working with independently collected clinical datasets, and it bounds the cross-cohort comparisons to the exploratory level.

#### Transcription and Model Biases

Although we used state-of-the-art speech-to-text models, transcript noise remains a potential source of bias. Mental disorders can alter speech production (eg, reduced energy and dysfluencies) [58], which may increase automatic speech recognition error rates that propagate to embeddings and clusters. We could not measure word or character error rates on our recordings, because no human reference transcripts were collected for any of the 4 cohorts. Published evaluations of the Whisper family report strong recognition accuracy for French, Italian, and Spanish and a higher error rate for Mandarin Chinese [50]. A further concern is differential error. If transcription quality varies with clinical status, transcription error could itself contribute to the associations between cluster membership and the clinical scales. We have no human reference transcripts to test this directly, and because recognition is weakest for Chinese, the Chinese cohort warrants the most caution here. Creating reference transcripts for a small subset would permit direct error-rate measurement and is a worthwhile direction for future work. Additionally, the language models used to embed transcripts and generate cluster descriptions may encode cultural or demographic biases [59]. LLMs also differ systematically in the values they prioritize in the text they generate [60]. As all cluster descriptions in this study were generated by a single model, Qwen3-14B, they may reflect that model’s value priorities rather than entirely neutral summaries of the transcripts.

#### Clinical Validation of Cluster Descriptions

The cluster descriptions are an interpretability layer, and this study makes no claim about their clinical accuracy, completeness, or relevance. Establishing that would require independent, blinded evaluation by multiple clinicians with formal interrater reliability (Cohen κ or the intraclass correlation coefficient), a distinct line of work. A board-certified psychiatrist read the descriptions as a coherence check. As all statistical associations are computed from cluster membership rather than from the descriptions, the findings do not depend on such clinical evaluation. The clusters that the pipeline recovers separate validated clinical scales in the French, Italian, and Chinese cohorts, and they are formed from the transcripts alone, without the clinical scores. The grounding of the descriptions in their transcripts (faithfulness), a comparison of each summary against its source text rather than a clinical judgment, is reported in Multimedia Appendix 9, where 39 of 40 descriptions assert no content that their transcripts do not support and their stability across transcript sampling, repeated generation, and prompt wording in Multimedia Appendix 6, where they prove highly reproducible.

### Comparison With Prior Work

#### Exploratory Cross-Cohort Observations

Across all 4 cohorts, clusters with higher depression or suicide risk scores consistently contained descriptions of negative emotional states, functional impairment, and disrupted relationships, while lower-risk clusters emphasized positive coping strategies, supportive relationships, and engagement in meaningful activities. We report this recurrence as a preliminary cross-cohort observation, not as evidence of cross-cultural generalizability, because the 4 datasets differ in language, population, clinical instruments, and interview questions; whether these markers generalize across linguistic and cultural contexts is a hypothesis for future studies with parallel data-collection protocols.

The descriptions also surfaced content specific to individual cohorts. In the Italian cohort, participants expressed strong regional identity and affiliation with Naples. In the Chinese cohort, family communication challenges and feelings of inadequacy featured prominently in higher-risk clusters. These cases show the pipeline captures cohort-specific themes a generic analysis might miss. Whether such content reflects broader cultural patterns or the cohorts’ particular recruitment and interview design cannot be determined from these nonparallel datasets, and would require dedicated cross-cultural studies with parallel protocols [18].

#### Risk and Protective Topics for Depression

We compared our generated topic descriptions with known risk and protective topics for depression studied in psychiatry research. A psychiatrist and a researcher in natural language processing reviewed the generated descriptions to relate them to risk and protective topics established in the literature.

Several clusters recapitulate established risk topics. Clusters with sleep disturbance or fatigue align with meta-analyses showing a bidirectional link between insomnia and depressive morbidity [61]. Stressor-laden clusters (stress due to unemployment or financial difficulties) track established longitudinal associations between adverse psychosocial work environments or job loss and increased depression risk [62]. Student-specific clusters may contain topics such as examination pressure that show associations between academic stressors and depressive symptoms [63].

Other clusters map onto protective topics. Clusters emphasizing arts participation and creative activity correspond to the WHO scoping review concluding that arts engagement contributes to prevention and symptom reduction in mental health [64]. Gardening and nature exposure clusters show consistency with evidence of small-to-moderate improvements in depressive symptoms from horticultural and green-space activities [65]. Mentions of holidays or travel appear predominantly in low-severity clusters and are congruent with longitudinal work showing short-lived but reliable gains in mood and well-being during vacations [66]. Finally, physical activity topics align with prospective meta-analyses, indicating that even subguideline levels of activity are associated with lower incident depression [67].

Taken together, the fine-grained topics generated by the language model are consistent with core psychiatric constructs established in the literature, a qualitative correspondence rather than a statistical one. We found risk topics (eg, related to sleep disruption, workload, and financial stressors) and protective topics (eg, related to arts, nature, holidays as short-term relief, and physical activity) in a multilingual, data-driven fashion that yields human-readable descriptions while scaling beyond manual thematic analysis.

#### Comparison With Keyword-Based Topic Modeling

Prior work on topic modeling in depression has relied on keyword-based approaches that surface topics as word lists. For instance, the study by Zhang et al [28] applied BERTopic to free-response speech in the RADAR-MDD dataset and identified 29 topics represented by keywords such as "daughter," "son," "parents," "hospital," "operation," "pain," "sleep," "tired," and "rest." While such keywords indicate broad thematic areas, they lack the semantic context and narrative coherence that clinicians use when interpreting patient discourse. That study is the closest precedent to our work. It analyzes a single English-language dataset, represents each topic as a keyword list, and relates the topics to Patient Health Questionnaire-8 depression scores. Our study builds on this line of work and broadens it to 4 cohorts in 4 languages, including a large general-population sample and 3 clinical cohorts, and to several clinical constructs (depression, anxiety, insomnia, fatigue, and suicide risk).

Our approach produces qualitatively different outputs and requires no manual post hoc categorization: the language model directly produces interpretable summaries, whereas keyword-based approaches typically require researchers to manually group and label topics. To make this concrete on our own data, we labeled the same clusters in 2 ways, with keyword labels from a standard keyword-based topic model (BERTopic, which ranks the words most distinctive to each cluster after removing common stopwords in each language) and with our language-model descriptions. We hold the clustering fixed, so every statistical association is unchanged and only what a reader can take from the label differs. The descriptions state the clinical character in a single readable sentence and disambiguate context a bare keyword cannot, whereas keyword lists mix the theme with idiosyncratic or generic words that carry no clinical content. For example, the most depressed cluster in the Chinese cohort carries the keyword label "feeling," especially, "think," "before," "now," "nothing," "seem," "do not want," "life," which conveys little clinical content, whereas its language-model description names physical health issues and emotional struggles such as "depression," "anxiety," and "loss of motivation." In the Spanish sadness-and-coping cohort, the description reports self-inflicted physical pain as a maladaptive coping strategy that the cluster’s keywords ("sad," "bad," "to feel," "sensation," "going out," "music," "week," "news," and "Madrid") do not surface. This contrast is not specific to our pipeline. Evaluations of topic models report that keyword-coherence metrics capture surface word statistics rather than the semantic quality a human reader needs, whereas language-model readings of topics align with human judgment [68,69]. The full side-by-side examples for every cohort, with the descriptions reproduced verbatim, are provided in Multimedia Appendix 8.

### Bridging Qualitative Research and Clinical Practice

Our introduction highlighted that manual thematic analysis, while clinically valuable, is too resource-intensive for routine care [14]. The cost is concrete. Full thematic analysis of a single qualitative interview study can require more than 100 hours of researcher time [70], and reliable manual coding further depends on trained coders and repeated double-coding to reach acceptable agreement [71]. The present study shows that language models can generate cluster descriptions consistent with core psychiatric constructs identified in labor-intensive qualitative research. For instance, our clusters containing sleep disturbance, emotional dysregulation, and impaired functioning align with the phenomenological dimensions of depression (altered temporal experience, embodied distress, and existential emptiness) identified through expert consensus [14].

This convergence indicates that automated methods can help bridge the gap between what qualitative analysis reveals as clinically important and what clinicians can feasibly assess at scale. Where the expert-consensus approach to identifying clinically validated themes required collaborative workshops and international expert panels [14], our pipeline recovered comparable thematic structure automatically, across 4 languages and thousands of transcripts, without trained coders. It performs the labor-intensive early stages of thematic analysis—familiarization with the material, initial coding, and theme generation—in a single automated pass, while validation and clinical interpretation remain with the clinician. On our own data, the gain in speed and cost is concrete. On a single NVIDIA A100 GPU, the pipeline clustered and labeled a French interview question in minutes of compute, at a cloud-equivalent cost under US $1. The familiarization, initial coding, and theme-generation stages it automates would take an estimated 90 to 170 analyst-hours by hand, and roughly double that under the independent double-coding that reliable analysis requires [71]. Across all 4 cohorts, the estimated manual effort runs to several hundred analyst-hours (Multimedia Appendix 10). This is an illustrative comparison of scale rather than a claim of equivalence. The pipeline is an automated topic-modeling procedure of embedding, clustering, and language-model description, and it accelerates these early stages, not the interpretive coding and clinical judgment that a full manual thematic analysis brings. This pattern matches the broader literature: generative-AI thematic analysis reproduced most human-derived themes while reducing analyst time by about 97% [72], with substantial reliability reported for automated content coding [73]. Blinded comparisons find LLMs comparable to human analysts when applying predefined codes, with low hallucination rates [74] and language-model theme summaries reaching substantial agreement with human-derived themes [75]. Our approach identified clinically relevant content from speech records across diverse cohorts. The language model generated meaningful syntheses using clinically agnostic prompts, without pre-established theory or domain-specific information.

However, automated methods complement rather than replace clinical judgment. Expert clinical judgment remains essential for contextualizing algorithmically discovered patterns and for distinguishing clinically actionable insights from statistical artifacts.

### Potential Clinical Applications

This study establishes the methodological foundations. The clinical applications below are future possibilities. Our analyses show that cluster membership is associated with validated clinical scales in the French, Italian, and Chinese cohorts, but they do not establish screening validity, diagnostic accuracy, treatment-planning utility, or readiness for clinical decision support. Each of the directions below would require dedicated prospective validation. First, cluster-based analysis could in principle support screening and risk stratification: individuals whose speech patterns place them in high-severity clusters might be flagged for priority clinical review. Second, the fine-grained topic descriptions could inform personalized treatment planning: a patient whose cluster emphasizes academic stress and sleep disturbance may benefit from different interventions than one whose cluster highlights social isolation and hopelessness. Third, tracking cluster membership or distance-to-cluster-centroid over time could enable treatment monitoring, providing objective measures of thematic shifts during therapy.

Critically, such applications require interpretability. Clinicians need to understand why a patient was assigned to a particular cluster to trust and act on algorithmic recommendations. Our approach addresses this need by generating human-readable descriptions rather than opaque embeddings or keyword lists. This interpretability is essential for developing safe and trustworthy clinical decision support systems based on speech or language analysis. Future work should evaluate whether cluster descriptions improve clinician decision-making in controlled settings.

### Conclusions

Systematic analysis of spontaneous speech at scale has been impractical, and existing automated methods describe topics with keyword lists that miss clinical nuance. The pipeline we introduce automates the labor-intensive early stages of thematic analysis (familiarization, initial coding, and theme generation) on spontaneous speech in 4 languages, in minutes of single-GPU compute per interview question, at a cost under US $1. It uses a language model to render each cluster as a description a clinician can read directly. In the French, Italian, and Chinese cohorts, score-agnostic clusters were significantly associated with clinical scores, with moderate to large effects (for example, PHQ-9 η²=0.19, and Cramér V=0.74 for clinician-diagnosed depression). The association in the smaller Spanish cohort was exploratory. The descriptions that label the clusters stayed grounded in the transcripts and were reproducible.

Two findings carry practical weight. Question choice matters: a single question about current feelings and sleep separated clusters by symptom severity, whereas questions about past or future events did not. Sociodemographic factors shaped topic content independently of clinical status, a confound rarely examined in computational psychiatry, although the association between cluster membership and clinical scores survived adjustment for age, sex, and education.

As the 4 datasets are independent and were not collected in parallel, the cross-cohort patterns are preliminary rather than evidence of cross-cultural generalizability. Screening, treatment-planning, and decision-support uses remain to be established through dedicated prospective study. By making patient narrative analyzable at scale in readable language, the approach can augment, not replace, the clinician’s judgment.

## Supplementary material

10.2196/99185 Multimedia Appendix 1 Descriptive statistics and sample sizes for the 4 cohorts.

10.2196/99185 Multimedia Appendix 2 Covariate-adjusted confound analysis for the French general population cohort.

10.2196/99185 Multimedia Appendix 3 Effect sizes across interview questions and clinical scores for the 4 cohorts.

10.2196/99185 Multimedia Appendix 4 Clustering stability and sensitivity across random seeds and minimum cluster size, internal cluster-validity indices, and bootstrap CIs for the reported effect sizes.

10.2196/99185 Multimedia Appendix 5 Pipeline hyperparameters and the cluster-description prompt.

10.2196/99185 Multimedia Appendix 6 Stability of the language-model cluster descriptions across transcript sampling, generation seeds, and prompt wording.

10.2196/99185 Multimedia Appendix 7 Small-cohort Monte-Carlo permutation tests with expected cell counts.

10.2196/99185 Multimedia Appendix 8 Keyword labels vs language-model descriptions for the same clusters.

10.2196/99185 Multimedia Appendix 9 Faithfulness of the cluster descriptions to their transcripts.

10.2196/99185 Multimedia Appendix 10 Computational cost and comparison with manual thematic analysis.

## Abbreviations

AIS: Athens Insomnia Scale
BDI: Beck Depression Inventory
C-SSRS: Columbia Suicide Severity Rating Scale
*DSM*: *Diagnostic and Statistical Manual of Mental Disorders*
*DSM-5*: *Diagnostic and Statistical Manual of Mental Disorders* (Fifth Edition)
*DSM-IV*: *Diagnostic and Statistical Manual of Mental Disorders* (Fourth Edition)
GAD-7: Generalized Anxiety Disorder 7-item scale
HDBSCAN: Hierarchical Density-Based Spatial Clustering of Applications With Noise
LLM: large language model
MDD: major depressive disorder
MFI: Multidimensional Fatigue Inventory
MODMA: multi-modal open dataset for mental-disorder analysis
PHQ-9: Patient Health Questionnaire-9
UMAP: Uniform Manifold Approximation and Projection
VOCES: Spanish clinical cohort for suicide-risk research
