# Supplementary material for: Interpretable Topic Modeling of Spontaneous Speech in Depression Using Large Language Models: Multilingual Four-Cohort Study
Source: JMIR Ment Health. 2026 Aug 17;13:e99185. doi: 10.2196/99185 (PMC13480703; doi:10.2196/99185)
Supplement: Supplementary file 1 [file mental-v13-e99185-s001.pdf]

Descriptive statistics and sample sizes for the 4 cohorts

This appendix reports, for each cohort and interview question, the number of transcripts entering the clustering analysis and their length characteristics, together with the number of participants, recordings, and transcripts per cohort. It supports the manuscript "Interpretable Topic Modeling of Spontaneous Speech in Depression Using Large Language Models: A Multilingual Four-Cohort Study."

Transcript counts and length statistics

Cohort	Question	Transcripts	Sentences	Chars	Words	TTR	Noise %
French (general population)	Last 24 hours	1782	7.9	683.0	143.2	0.61	9.8
	Past negative event	1400	5.1	458.0	94.6	0.74	3.1
	Past positive event	1393	4.0	347.6	70.6	0.77	6.6
	Future negative event	1387	3.1	275.0	55.0	0.80	7.5
	Future positive event	1391	2.9	234.1	46.5	0.82	7.4
	Current feelings and sleep	1786	4.9	393.8	82.8	0.70	7.9
Italian (Androids)	Everyday life	110	25.7	2362.1	487.6	0.44	3.6
Chinese (MODMA)	Positive questions	52	2.6	243.4	11.2	0.63	0.0
	Negative questions	52	2.1	271.2	8.5	0.60	0.0
	Neutral questions	52	2.3	269.0	10.5	0.62	0.0
Spanish (VOCES)	Sadness and coping	58	33.1	1648.4	346.6	0.43	0.0
	Average day	78	28.6	1559.6	332.8	0.41	0.0
	Recent good news	55	19.3	1249.7	265.9	0.46	0.0

Table S1. Descriptive statistics across cohorts. Transcripts = number of transcripts; Sentences, Chars, and Words are mean counts per transcript; TTR = type-token ratio (unique tokens divided by total number of tokens); the Question column gives short task labels, and the corresponding interview questions are listed per cohort in the manuscript Recruitment section; Noise % = percentage of transcripts assigned to HDBSCAN noise and therefore excluded from the cluster-level statistical tests.

Participants, recordings, and transcripts

Cohort	Participants	Recordings or visits	Analyzed transcripts (reported task)	Content clusters
French (general population)	1338	1809 visits	1786 (feelings and sleep)	26
Italian (Androids)	116	228 recordings	110 (interview)	5
Chinese (MODMA)	52	52	52 (neutral)	4
Spanish (VOCES)	90	90	58 (sadness)	5

Table S2. Participants, recordings, and analyzed transcripts per cohort. The French cohort is analyzed per task, so each participant could contribute several transcripts (1 per interview question). The reported clustering for the question "Describe how you are feeling at the moment and how your sleep has been lately" used 1786 transcripts. The Italian cohort contributed 2 recording types (a spontaneous interview and a read-speech task). Only the interview task was analyzed. Spanish per-task counts vary by question because not all participants answered every question. Exclusion criteria: duplicate recordings and transcripts shorter than 20 characters were removed before clustering. HDBSCAN noise points were retained for description generation but excluded from the cluster-level statistical tests (noise proportions are reported in the table above).
