# Supplementary material for: Interpretable Topic Modeling of Spontaneous Speech in Depression Using Large Language Models: Multilingual Four-Cohort Study
Source: JMIR Ment Health. 2026 Aug 17;13:e99185. doi: 10.2196/99185 (PMC13480703; doi:10.2196/99185)
Supplement: Supplementary file 2 [file mental-v13-e99185-s002.pdf]

Covariate-adjusted confound analysis (French general population cohort)

This appendix reports a covariate-adjusted analysis testing whether the associations between cluster membership and clinical scores in the French general population cohort survive adjustment for age, sex, and education. It supports the manuscript "Interpretable Topic Modeling of Spontaneous Speech in Depression Using Large Language Models: A Multilingual Four-Cohort Study."

Purpose

Because language use varies with age, sex, and education independently of mental health, the association between cluster membership and clinical scores could be confounded by sociodemographic composition. A concrete concern is whether a "depression-associated" cluster composed predominantly of young adults reflects age rather than clinical content. We therefore tested whether the association between cluster membership and clinical scores survives adjustment for age, sex, and education, and whether it is robust to repeated visits.

Method

We ran this analysis on the French cohort question "Describe how you are feeling at the moment and how your sleep has been lately." We chose this question because, across the full grid of questions and clinical scores, it is the one whose clusters are most strongly and most consistently associated with clinical status (the largest cluster effect sizes for PHQ-9, GAD-7, BDI, AIS, and MFI; see Multimedia Appendix 3). It is therefore the question on which a demographic confound, if present, would most plausibly inflate the apparent association with clinical scores, and the natural one on which to test whether that association survives adjustment. Excluding noise points, we fitted for each clinical score 3 ordinary least-squares models: (1) score on cluster membership only; (2) score on covariates only (age, sex, education); and (3) score on cluster membership plus covariates. We report the partial eta-squared of cluster before and after adjustment and a joint Wald test of the cluster terms using standard errors clustered by participant to account for repeated visits. As a sensitivity analysis we refit all models restricting the data to 1 visit per participant (first visit). For the adjusted cluster partial eta-squared we report nonparametric percentile bootstrap 95% CIs (2000 resamples), resampling participants with replacement and taking all of their transcripts, the same resampling unit used for the bootstrap intervals in Multimedia Appendix 4. The analysis sample was 1645 transcripts from 1228 participants.

Results

For every score, the cluster effect is essentially unchanged by adjustment, and cluster membership explains more variance than demographics. For PHQ-9, demographics alone explained 5.3% of variance and cluster membership alone 23.2%. The cluster partial η^2 moved only from 0.232

(unadjusted) to 0.220 (adjusted, 95% CI 0.188 to 0.279), and the joint test of cluster membership (with participant-clustered standard errors) remained highly significant (F=12.9, P<.001).

**Score**	R^2 **covariates**	**Partial η^2 cluster (unadj.)**	**Partial η^2 cluster (adj., 95% CI)**	**Patient-clustered Wald**
PHQ-9	0.053	0.232	0.220 (0.188 to 0.279)	F=12.9, P<.001
GAD-7	0.046	0.185	0.168 (0.143 to 0.221)	F=9.3, P<.001
BDI	0.024	0.199	0.193 (0.161 to 0.251)	F=10.9, P<.001
AIS	0.013	0.177	0.172 (0.146 to 0.225)	F=11.6, P<.001
MFI	0.048	0.172	0.164 (0.141 to 0.215)	F=11.9, P<.001

Table S1. Covariate-adjusted confound analysis for the French general population cohort (the feelings-and-sleep question). Covariates are age, sex, and education, and R^2 covariates is the variance they explain alone. Partial η^2 of cluster membership, the proportion of score variance attributable to it after accounting for the covariates, is reported before and after adjustment. Unadjusted, it equals the variance cluster membership explains on its own. The joint Wald test of the cluster terms uses standard errors clustered by participant.

The one-visit-per-participant sensitivity analysis (n=1228) gave near-identical estimates (for example, PHQ-9 adjusted partial η^2 0.210, F=12.2, P<.001), confirming the association is not driven by participants with repeat visits.

Interpretation

The association between cluster membership and clinical scores is largely independent of age, sex, and education: demographics account for a small share of score variance (5% for PHQ-9), whereas cluster membership remains strongly and significantly associated after adjustment. Demographics do matter, since age and sex are themselves independently associated with cluster membership, as the study reports. The association between cluster membership and clinical scores is nonetheless not an artifact of demographic composition. We therefore report the confound as a genuine but partial contributor, not as one that accounts for the clinical findings entirely.

Why this analysis is reported for the French cohort only

The French general population cohort is the one cohort in which a covariate-adjusted analysis is both necessary and feasible. It is necessary because its demographics vary widely and are themselves associated with the clinical scores (for example, age differs significantly across the cohort), so demographic confounding is a genuine concern. It is feasible because the cohort is large enough (more than 1600 transcripts) to fit a stable multivariable model. The clinical cohorts differ on both counts. In the Italian cohort, controls were matched to cases on age, sex, and education at recruitment. In the Chinese cohort, these demographic variables do not differ significantly between groups (manuscript Table 1). In both, demographic confounding is therefore constrained rather than something an adjusted model needs to remove. Their sample sizes (n=52 to 116), combined with several clusters, are also too small to support a stable covariate-adjusted model of clinical status on cluster membership plus demographics. The Spanish cohort, where age does differ between groups, has a suicide-risk association that is already reported as exploratory and sensitive to the

clustering seed (Multimedia Appendix 4), so an adjusted model there would not be trustworthy either. We therefore fit and interpret covariate-adjusted models for the French cohort only. For the clinical cohorts, we rely instead on their recruitment design: matched controls in the Italian cohort and demographically balanced groups in the Chinese cohort.

Supporting figure

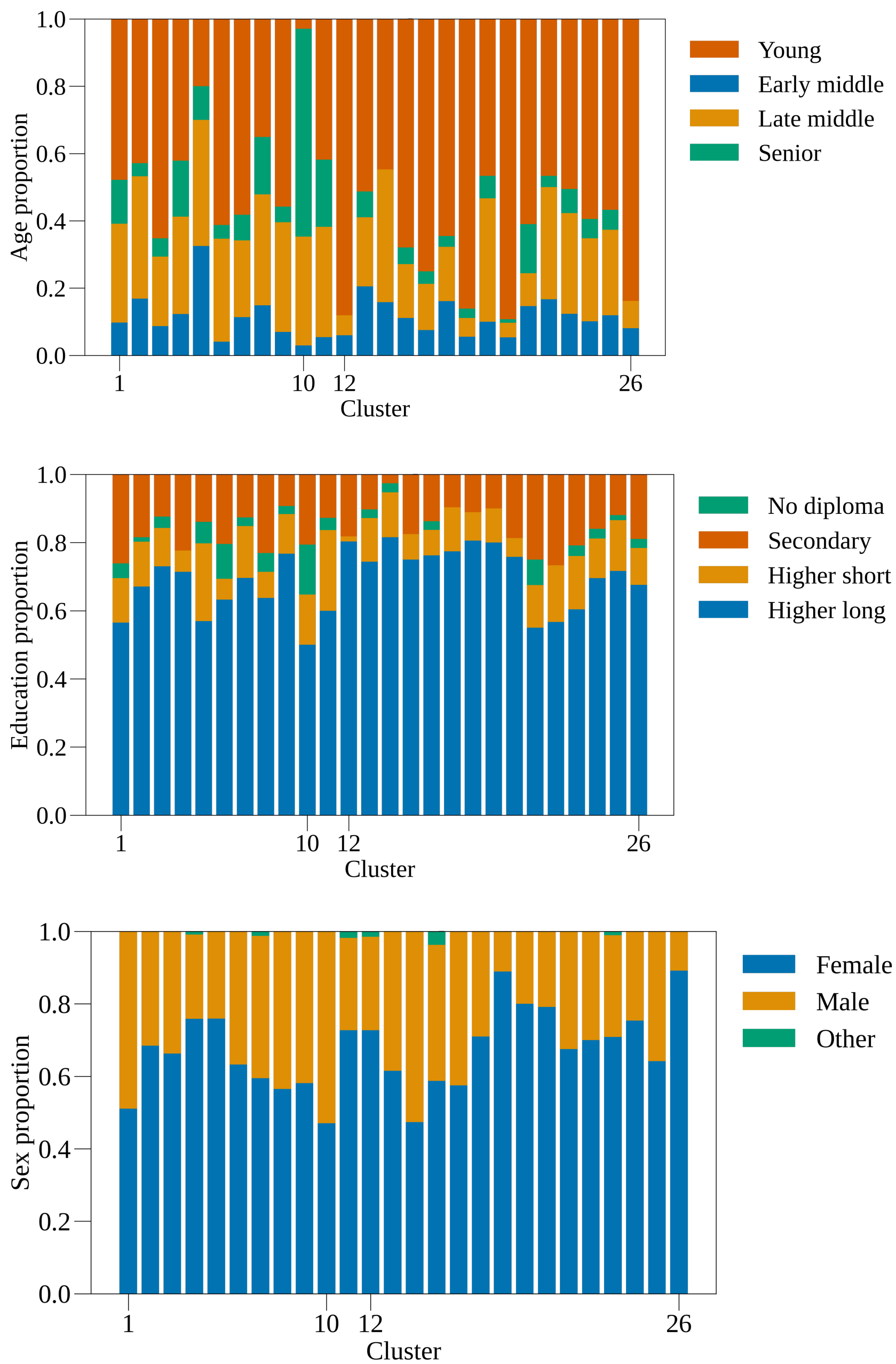

Figure S1. Confounding effect of PHQ-9 with sociodemographics (age, education, and sex). Transcripts are answers to the feelings-and-sleep question in the French general population cohort. Cluster membership is significantly associated with age (η^2=0.16), education (Cramér V=0.16), and sex (Cramér V=0.18), all P<.001.
