# Supplementary material for: Interpretable Topic Modeling of Spontaneous Speech in Depression Using Large Language Models: Multilingual Four-Cohort Study
Source: JMIR Ment Health. 2026 Aug 17;13:e99185. doi: 10.2196/99185 (PMC13480703; doi:10.2196/99185)
Supplement: Supplementary file 3 [file mental-v13-e99185-s003.pdf]

Effect sizes across interview questions and clinical scores

This appendix reports the full grid of association effect sizes between cluster membership and clinical or sociodemographic variables, across interview questions, for each of the 4 cohorts. It supports the manuscript "Interpretable Topic Modeling of Spontaneous Speech in Depression Using Large Language Models: A Multilingual Four-Cohort Study."

Two distinct association measures are reported depending on the variable type and should not be read on a single common scale: eta-squared (η^2, derived from Kruskal-Wallis tests) quantifies the association between cluster membership and a continuous score (eg, PHQ-9, GAD-7, age), whereas Cramér V (derived from chi-square tests) quantifies the association with a categorical outcome (eg, DSM-5 depression diagnosis, MDD diagnosis, C-SSRS suicide-risk category, sex, education level). Each statistic has its own colorbar, and cell color encodes the Cohen effect-size category. For η^2 the bands are negligible ($<$0.01), small (0.01 to 0.06), medium (0.06 to 0.14), and large ($\geq$0.14). For Cramér V the bands are negligible ($<$0.10), small (0.10 to 0.30), medium (0.30 to 0.50), and large ($\geq$0.50). The exact effect size is printed in each cell. Coloring by category means the same color denotes the same band in every figure, while η^2 and Cramér V remain on separate colorbars because they are different statistics. These effect sizes are exploratory rather than confirmatory, as described in the manuscript Methods. In every heatmap, a blank cell indicates no statistically significant difference between clusters after the joint Benjamini-Hochberg false-discovery-rate adjustment across the grid (manuscript Methods). A short observation follows each heatmap. The 95% CIs for the effect sizes reported in the manuscript, and their stability across clustering seeds, are reported in Multimedia Appendix 4.

French general population cohort

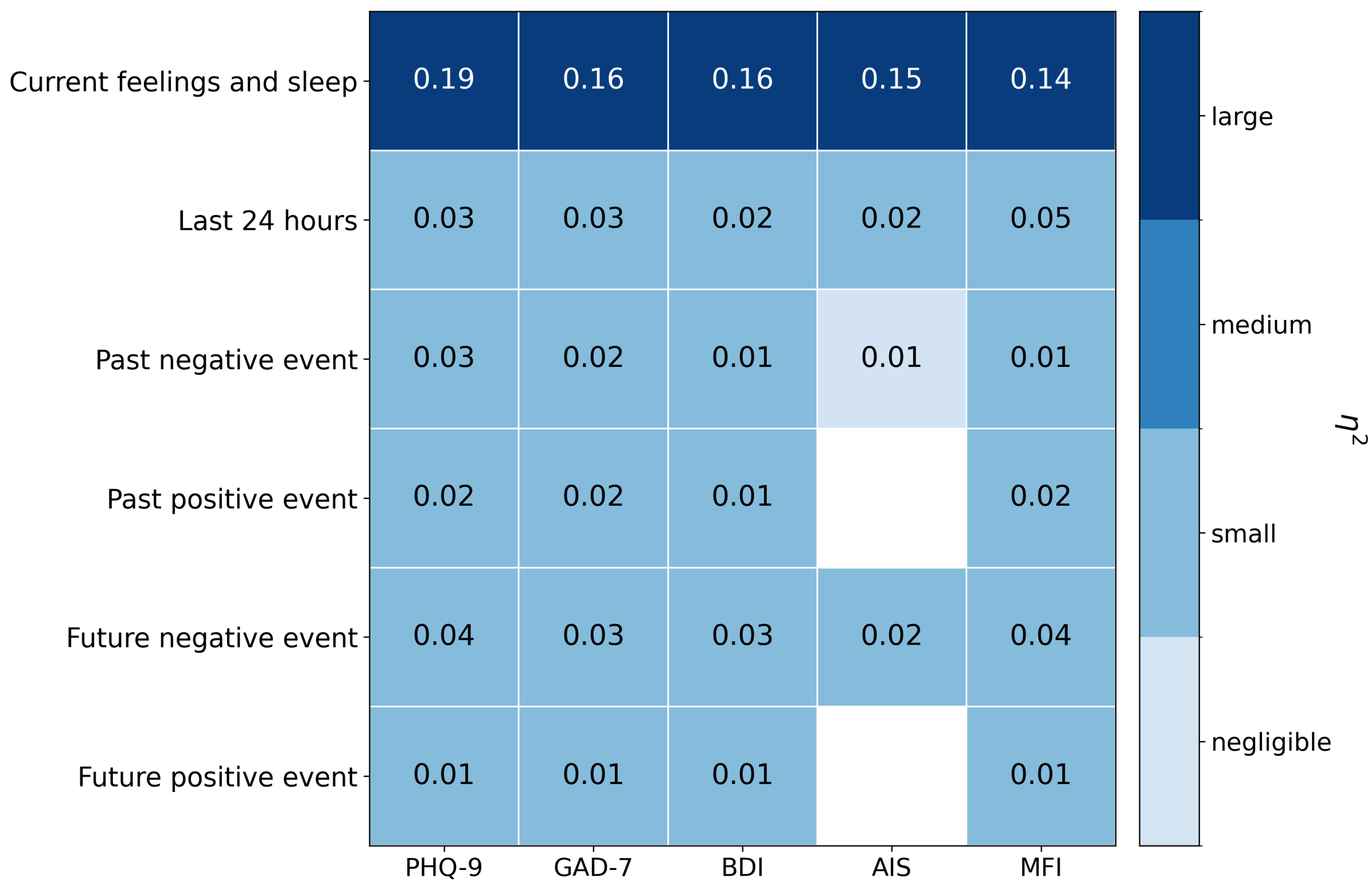

Figure S1. French general population: effect size (η^2, Kruskal-Wallis tests on continuous scores) across tasks and clinical scores.

Observation. The feelings-and-sleep question dominates this grid. It carries the only substantial effects, peaking at PHQ-9 (η^2=0.19) and staying in the 0.14 to 0.16 band for GAD-7, BDI, AIS, and MFI. Every other question is smaller, at or below η^2=0.05 across all 5 scores and at or below 0.04 on the past- and future-event questions, and several event-based cells are blank (no significant cluster difference after correction). A single question about how a person feels and sleeps right now is therefore the one worth asking when the goal is to separate clusters by depression, anxiety, insomnia, and fatigue severity.

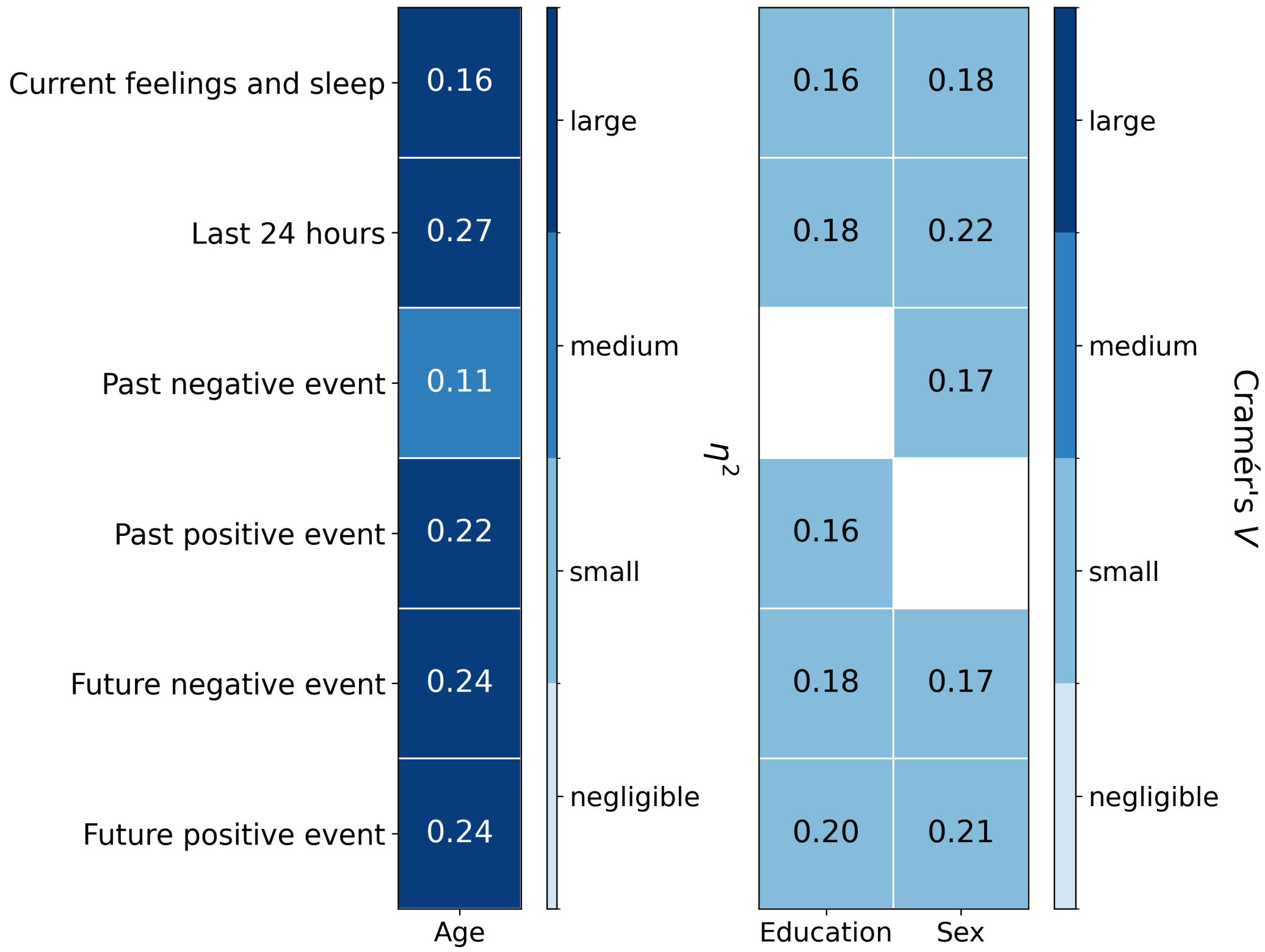

Figure S2. French general population: effect size (η^2 from Kruskal-Wallis tests for continuous demographics such as age, and Cramér V from chi-square tests for categorical demographics such as sex and education) across tasks and demographic variables.

Observation. Age is the strongest sociodemographic association here. It is largest for the "last 24 hours" question (η^2=0.27), and the positive-past and future-event questions are also substantial (η^2 0.22 to 0.24), while the past-negative-event question is weaker (η^2=0.11). Education and sex associations are present but smaller (Cramér V around 0.16 to 0.22), and a few cells are blank, for example education for the past-negative-event question and sex for the past-positive-event question. Topic content therefore tracks normal life stage, especially age, across almost every question, so demographic structure must be accounted for when reading clinical clusters. The covariate-adjusted analysis in Multimedia Appendix 2 shows the associations between cluster membership and clinical scores survive adjustment for these demographics.

Italian clinical cohort (Androids)

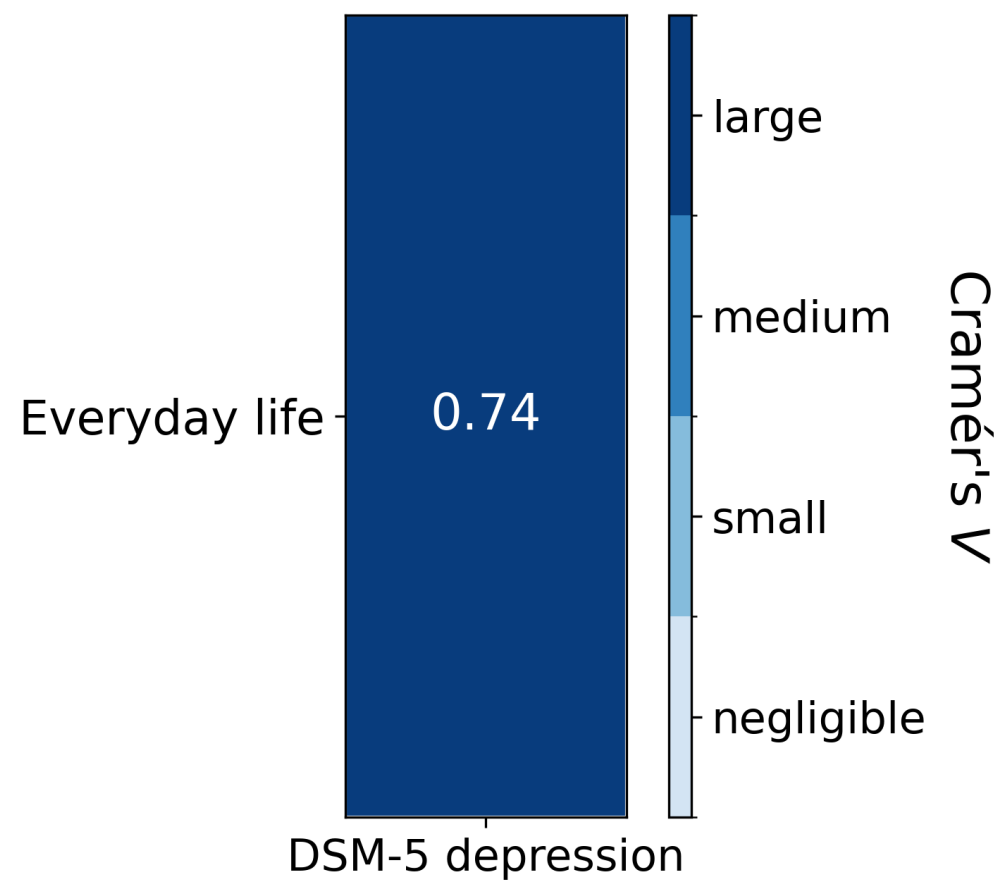

Figure S3. Effect size across tasks and clinical scores for the Androids cohort.

Observation. The single everyday-life question separates clusters by DSM-5 depression status strongly (Cramér V=0.74). This is the largest clinical association in the appendix, and it shows that even one open question about everyday life can yield clusters that map closely onto clinician-diagnosed depression.

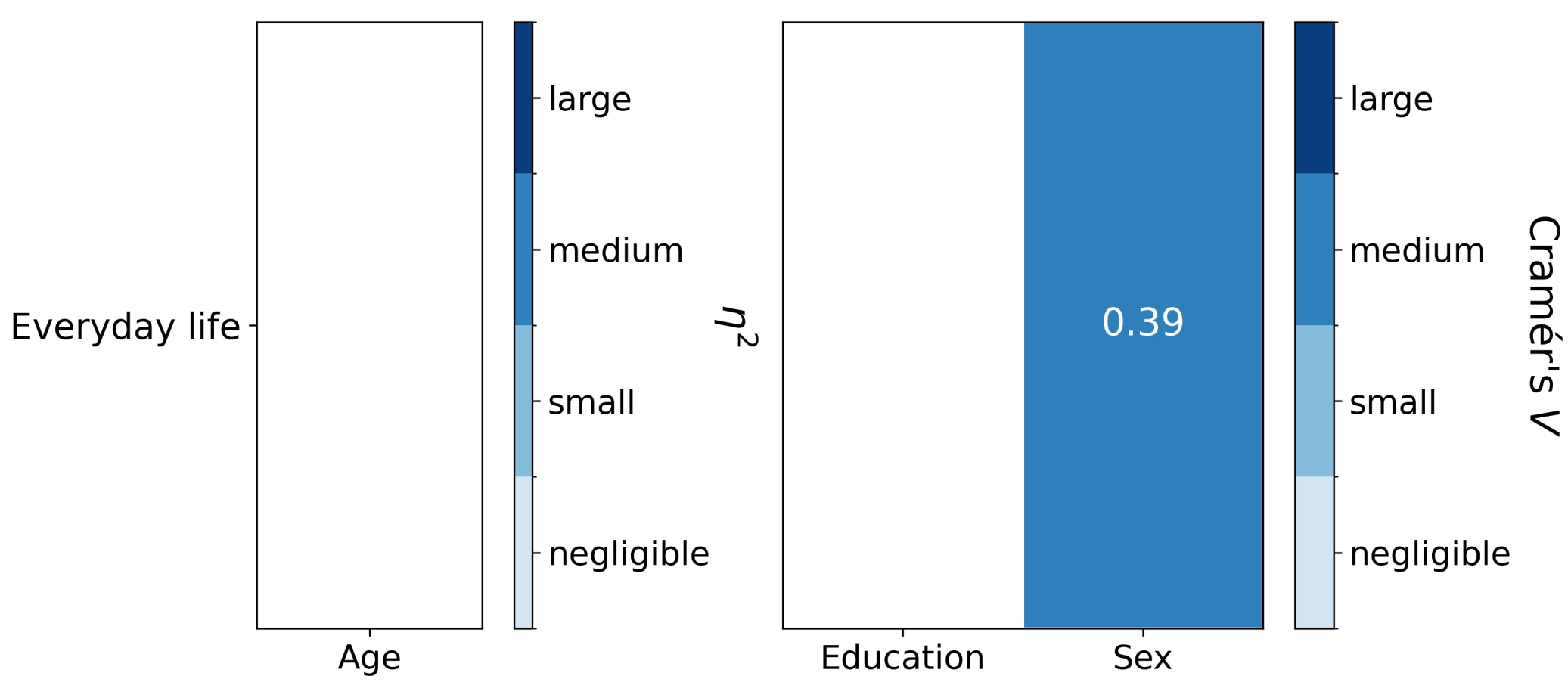

Figure S4. Effect size across tasks and sociodemographics for the Androids cohort.

Observation. In this cohort only sex is associated with cluster membership (Cramér V=0.39), while age and education show no significant difference (blank cells). The strong depression association in the previous figure is therefore not mirrored by a broad demographic one, so the depression-related clustering here is not simply tracking age or education.

Chinese clinical cohort (MODMA)

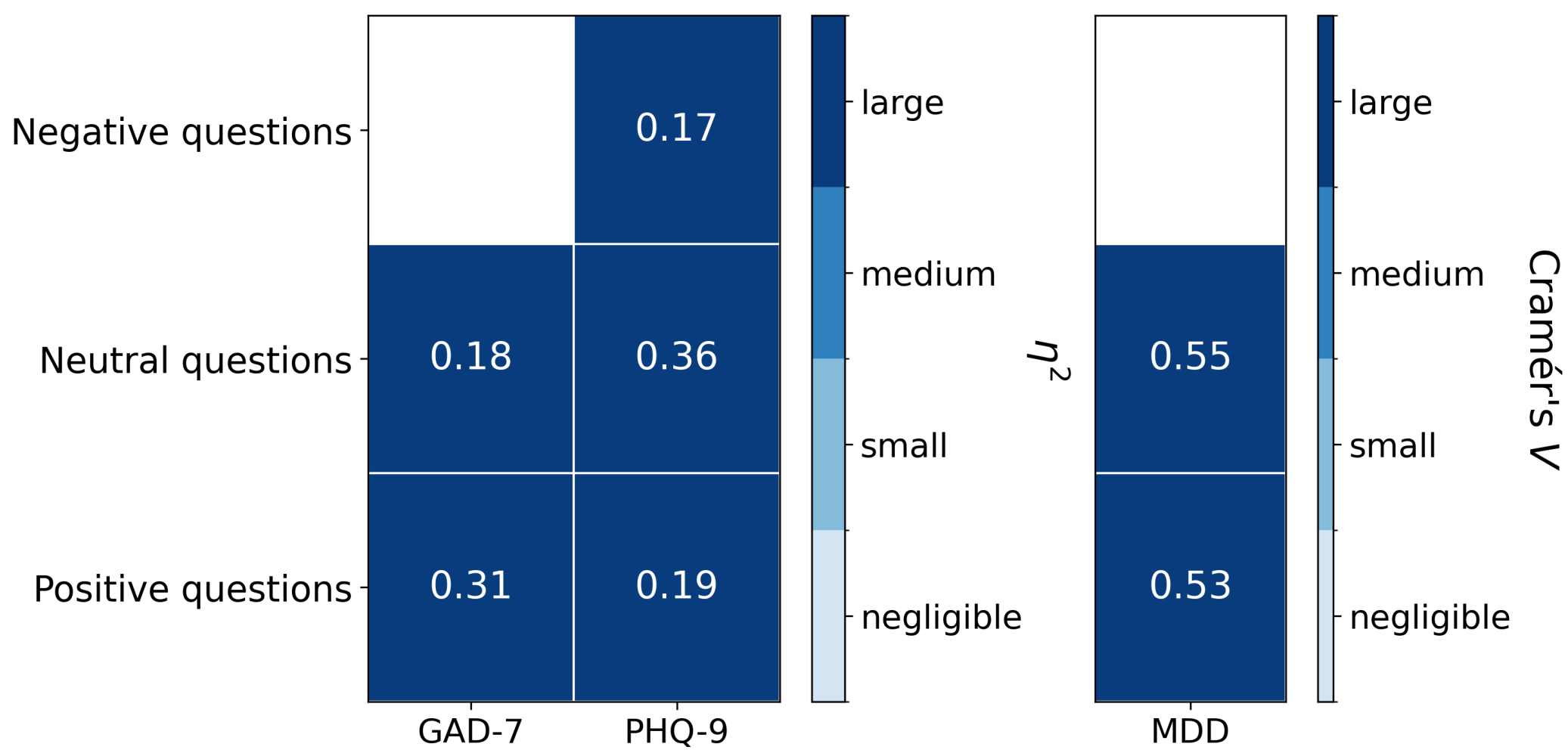

Figure S5. Effect size across tasks and clinical scores for the MODMA cohort.

Observation. MDD diagnosis is the outcome most strongly associated with cluster membership, with a moderate-to-large association for both the neutral and positive question groups (Cramér V=0.55 and 0.53). Among the continuous scores, PHQ-9 peaks at the neutral questions (η^2=0.36) and GAD-7 at the positive questions (η^2=0.31), while the negative questions are weaker and the GAD-7 cell there is blank. In this small cohort, neutral and positive questions therefore separate depression better than negative questions.

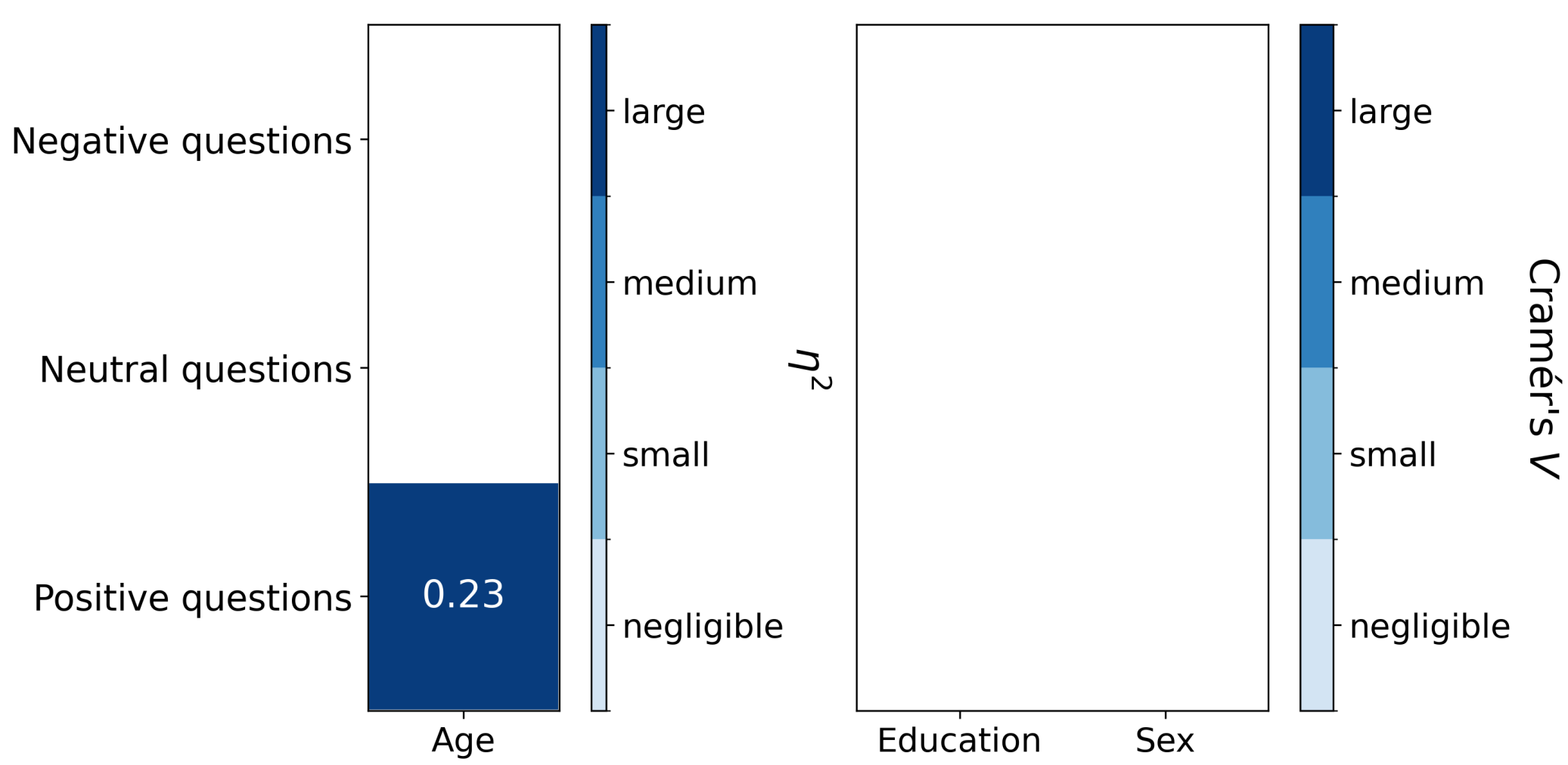

Figure S6. Effect size across tasks and sociodemographics for the MODMA cohort.

Observation. The only significant sociodemographic association is age for the positive question group (η^2=0.23). Education and sex show no significant differences (blank cells), and the other question groups are also blank for age. Demographic structure is therefore sparse in this cohort, so the MDD association in the previous figure is largely independent of the recorded demographics.

Spanish clinical cohort (VOCES)

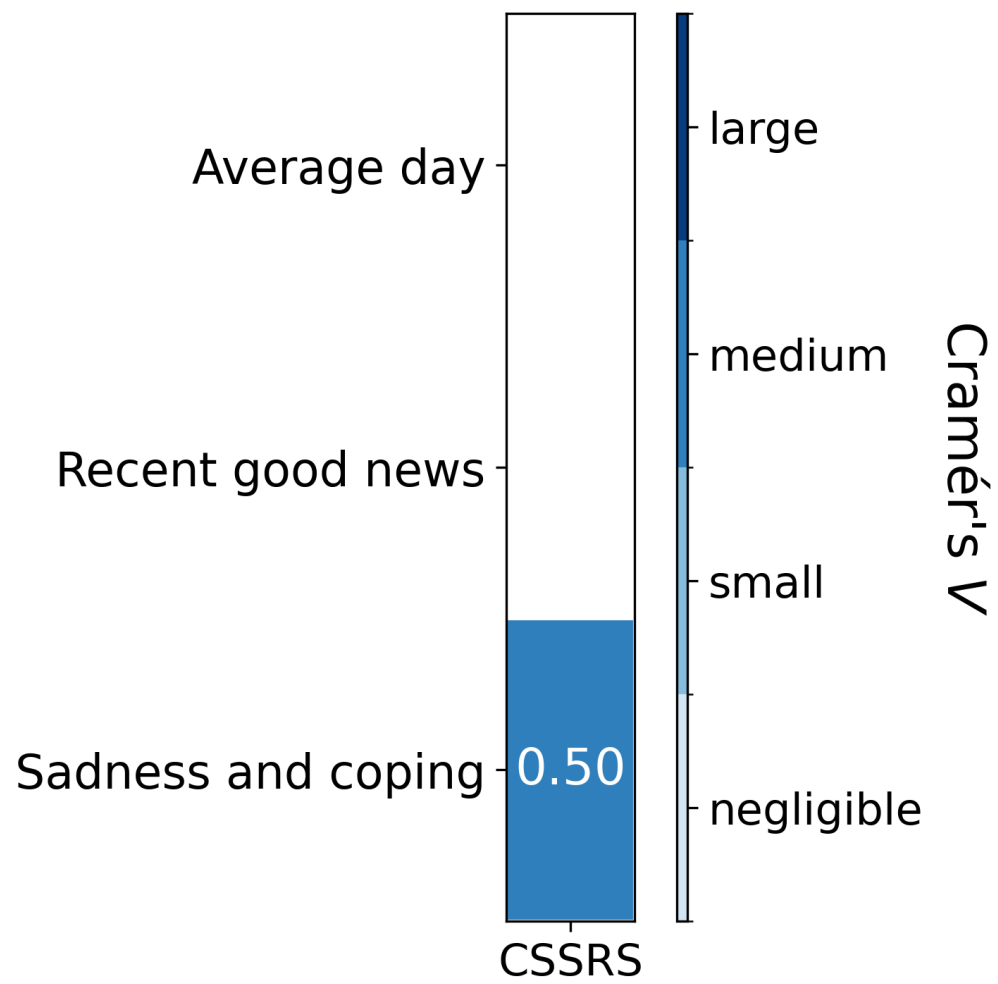

Figure S7. Effect size across tasks and clinical scores for the VOCES cohort.

Observation. Only the "sadness and coping" question shows a significant association with C-SSRS suicide-risk class (Cramér V=0.50 for the run displayed), while "average day" and "recent good news" are blank. This single association is unstable across clustering seeds in this smallest cohort (Multimedia Appendix 4), so we treat it as exploratory and report it by its seed-averaged value (mean Cramér V=0.27). The implication is a hypothesis to test in a larger sample rather than a confirmed effect.

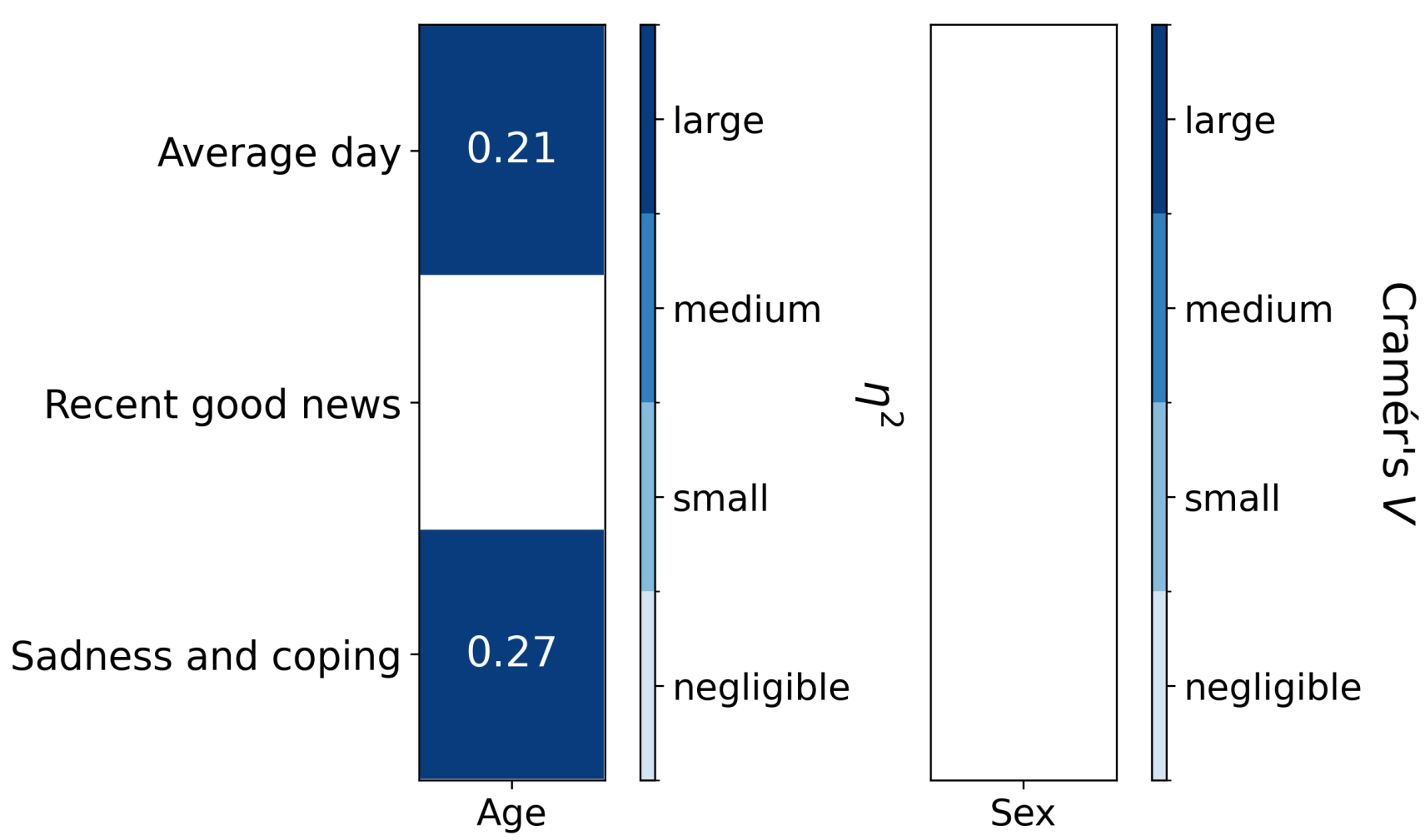

Figure S8. Effect size across tasks and sociodemographics for the VOCES cohort.

Observation. Age is associated with cluster membership for the "average day" and "sadness and coping" questions (η^2=0.21 and 0.27), while sex shows no significant difference at any question (blank cells). As in the other cohorts, age tracks topic content even in this small sample, which is worth keeping in view given that the suicide-risk association in the previous figure is exploratory.
