# Supplementary material for: Interpretable Topic Modeling of Spontaneous Speech in Depression Using Large Language Models: Multilingual Four-Cohort Study
Source: JMIR Ment Health. 2026 Aug 17;13:e99185. doi: 10.2196/99185 (PMC13480703; doi:10.2196/99185)
Supplement: Supplementary file 4 [file mental-v13-e99185-s004.pdf]

Robustness of the clustering and its clinical associations

This appendix subjects the clustering and the clinical associations it supports to a battery of robustness checks, so that no reported result rests on a single arbitrary choice in the pipeline. We test 4 properties: the stability of the clustering across the random seed of the dimensionality reduction and across the HDBSCAN minimum cluster size, its sensitivity to the dimensionality reduction itself (the number of components and the choice of reduction method), the internal validity of the recovered clusters, and the sampling uncertainty of every reported effect size. The seed-stability and minimum-cluster-size checks cover every interview question in each cohort, with particular attention to the small clinical cohorts where few transcripts are available. The dimensionality-reduction and internal-validity checks focus on the question each cohort's reported association rests on, and the bootstrap intervals cover every effect size the manuscript reports. The 4 sections below are self-contained: each states what was varied, reports the result in a table, and gives the reading. The stability of the language-model descriptions themselves, across repeated generation and resampled transcripts, is a separate question addressed in Multimedia Appendix 6. This appendix supports the manuscript "Interpretable Topic Modeling of Spontaneous Speech in Depression Using Large Language Models: A Multilingual Four-Cohort Study."

Stability across random seeds and minimum cluster size

This analysis isolates the main source of stochasticity in the pipeline, the dimensionality-reduction step: UMAP is stochastic, so different random seeds yield slightly different low-dimensional embeddings and therefore different clusterings (HDBSCAN is deterministic given its input). For every question analyzed in the study (6 for French, 1 for Italian, 3 for Chinese, and 3 for Spanish), we reran the dimensionality reduction and HDBSCAN clustering across 10 random seeds. Within each cohort we track its primary clinical outcome, the one its reported association rests on (PHQ-9 for French, DSM-5 depression status for Italian, MDD diagnosis for Chinese, C-SSRS risk class for Spanish), so the stability we report is the stability of the result the study draws on. For each run we recorded the number of clusters, the noise proportion, and the effect size (Kruskal-Wallis eta-squared for the continuous French score; Cramér V of cluster by clinical status for the clinical cohorts), excluding noise points. We report the mean pairwise adjusted Rand index (ARI) across runs as a measure of partition stability. Across cohorts the seed-averaged effect sizes are close to the reported ones and usually slightly higher, because reclustering assigns to clusters some points that the primary run had left as noise.

We separately swept the HDBSCAN minimum cluster size from half to twice its default value, with a floor of 3 transcripts because a minimum size of 2 produces degenerate clusters of near-pairs. The default minimum cluster size is 30 transcripts for the French cohort and one-tenth of the cohort (5 to 11 transcripts) for the smaller clinical cohorts, matching the published pipeline (Multimedia Appendix 5).

The seed analysis supports 2 conclusions. Within each cohort, the primary effect size is stable across random seeds: its coefficient of variation is small (1% for the Italian interview, 6% for the French feelings-and-sleep question, 2 to 8% for the Chinese questions), and partitions are highly stable across seeds in every case but one (mean pairwise ARI 0.63 to 0.94; only the Chinese neutral-questions partition, at 0.63, is moderate). The analysis also reproduces, without relying on a single

Task	n	Default min. size	Clusters (range)	Mean pairwise ARI	Effect size (mean ± SD)	CV	Effect over min-size sweep
French general population (PHQ-9, Kruskal-Wallis η^2)							
Last 24 hours	1782	30	23-26	0.82	0.034 ± 0.005	14%	0.023-0.042
Past negative event	1400	30	15-19	0.84	0.034 ± 0.005	15%	0.030-0.041
Past positive event	1393	30	15-19	0.85	0.014 ± 0.003	20%	0.010-0.023
Future negative event	1387	30	15-19	0.84	0.044 ± 0.004	8%	0.035-0.063
Future positive event	1391	30	19-21	0.94	0.019 ± 0.001	7%	0.015-0.025
Current feelings and sleep	1786	30	24-27	0.78	0.199 ± 0.012	6%	0.191-0.220
Italian clinical, Androids (DSM-5 depression, Cramér V)							
Everyday life	110	11	4-6	0.78	0.85 ± 0.01	1%	0.83-0.88
Chinese clinical, MODMA (MDD diagnosis, Cramér V)							
Neutral questions	52	5	3-6	0.63	0.57 ± 0.05	8%	0.43-0.64
Positive questions	52	5	2-4	0.89	0.37 ± 0.01	2%	0.37
Negative questions	52	5	4-6	0.78	0.51 ± 0.04	7%	0.37-0.55
Spanish clinical, VOCES (C-SSRS risk class, Cramér V)							
Sadness and coping	58	5	2-4	0.76	0.27 ± 0.09	34%	0.22-0.42
Average day	78	7	3-4	0.92	0.15 ± 0.01	5%	0.12-0.15
Recent good news	55	5	4-5	0.88	0.53 ± 0.02	4%	0.51

Table S1. Stability of cluster count, partition, and effect size across 10 random seeds, for every question analyzed in each cohort. ARI = mean pairwise adjusted Rand index between runs (partition stability); CV = coefficient of variation of the effect size across seeds. The effect size is the Kruskal-Wallis eta-squared (French, PHQ-9) or Cramér V of cluster by clinical status (clinical cohorts). The last column gives the range of the effect size as the HDBSCAN minimum cluster size is swept from half (floored at 3, since a size of 2 yields degenerate near-pair clusters) to twice its default.

seed, the study's finding that only certain interview questions (notably feelings-and-sleep in the French cohort) yield clusters associated with clinical status.

French. Only the "feelings and sleep" question produces a substantial PHQ-9 association (η^2=0.20), and it is stable (CV 6%, ARI 0.78). Every other French question produces a small PHQ-9 effect ($\eta^2 \leq 0.05$). These small effects are themselves stable in absolute terms (SD 0.001 to 0.005). Their larger coefficients of variation come only from dividing a small mean, so the effect is stably near zero rather than unstable. The seed analysis therefore confirms that the feelings-and-sleep question is genuinely the one whose clusters are associated with the PHQ-9 score rather than an artifact of a single favorable seed.

Italian and Chinese. The depression associations are moderate to large and stable across seeds and across the minimum-cluster-size sweep. The Chinese MDD association holds across all 3 question types (mean Cramér V 0.37 to 0.57, CV ≤8%).

Spanish. Stability is task-dependent, and the smallest-cohort task is the most fragile. The "sadness and coping" question, on which the study centers its exploratory suicide-risk observation, is the seed-sensitive one (CV 34%, effect size 0.16 to 0.44 across the 10 seeds, and 0.22 to 0.42 across the minimum-cluster-size sweep). We keep that finding exploratory and report it by its seed-averaged value (mean Cramér V=0.27). The published partition for this question has 5 clusters (the basis for the bootstrap interval below and for Multimedia Appendices 5 and 9), whereas the seeded reruns give 2 to 4. This divergence in cluster count is itself part of the instability and a further reason the suicide-risk association is exploratory. The "average day" and "recent good news" questions are more stable across seeds, but neither shows a significant suicide-risk association (Multimedia Appendix 3), so no claim rests on them.

Minimum-cluster-size sweep. This sweep examines whether, in the small clinical cohorts, clusters of about a tenth of the sample could be unstable. The minimum cluster size sets the granularity of the partition, so changing it alters the number of clusters and, with them, the effect-size metric. Finer settings produce more and smaller clusters, which inflates the Cramér V at small sample sizes. The sweep therefore tests whether the qualitative conclusions hold across granularity, not whether the effect-size magnitude is constant. For the French and Italian cohorts the magnitude is stable regardless (French feelings-and-sleep η^2 0.19 to 0.22, Italian DSM-5 depression Cramér V 0.83 to 0.88). The small clinical cohorts keep at least a moderate association throughout (for example, Chinese MDD Cramér V 0.43 to 0.64), with wider ranges that reflect both genuine fragility and the metric's dependence on cluster count, consistent with their seed sensitivity and with treating the Spanish suicide-risk association as exploratory.

Sensitivity to the dimensionality reduction

We varied the dimensionality of the UMAP reduction that HDBSCAN clusters in, sweeping its target dimensionality over 2, 3, 5, 8, and 10 dimensions (5 is the published value) and averaging the effect size over 5 seeds at each setting, on the principal question of each cohort. To test the choice of method rather than its dimensionality, we reran the principal question of each cohort with the UMAP reduction replaced by PCA (linear) and by t-SNE, each reducing the embeddings to the same 5 dimensions on its own, with the downstream HDBSCAN settings unchanged.

		**UMAP components**				
**Cohort (principal question)**	**Statistic**	**2**	**3**	**5**	**8**	**10**
French (feelings and sleep)	η^2	0.20	0.20	0.21	0.19	0.19
Italian (everyday life)	V	0.84	0.84	0.85	0.85	0.85
Chinese (neutral questions)	V	0.53	0.54	0.57	0.57	0.56
Spanish (sadness and coping)	V	0.29	0.23	0.27	0.24	0.28

Table S2. Effect size as the UMAP target dimensionality is varied (the published value is 5). Each entry is the mean over 5 random seeds. The clinical associations are essentially invariant to the number of components. The Spanish suicide-risk association stays weak and seed-variable (per-seed SD 0.08 to 0.12) at every dimensionality, consistent with the rest of this appendix.

The clinical associations are insensitive to the dimensionality of the reduction (Table S2): varying the UMAP target dimensionality from 2 to 10 leaves the French PHQ-9 effect at η^2 0.19 to 0.21, the Italian depression association at Cramér V 0.84 to 0.85, and the Chinese MDD association at V 0.53 to 0.57, while the Spanish suicide-risk association stays weak and seed-variable throughout. The choice of reduction method matters more than its dimensionality (Table S3). Replacing UMAP with linear PCA or with t-SNE, each reducing the embeddings to the same 5 dimensions on its own with the clustering settings unchanged, does not improve on the published UMAP association in any cohort: UMAP yields the strongest effect size for every cohort, and in the French and Italian cohorts, where it resolves about 26 and 4 to 6 clusters respectively, both alternatives collapse the data into 2 coarse clusters with weaker associations. The Spanish suicide-risk association stays weak under every reducer, consistent with the exploratory, seed-variable status we assign it throughout. This pattern is expected, because linear PCA and t-SNE do not preserve the local neighborhood density that HDBSCAN relies on, whereas UMAP is built to. A neighborhood-preserving nonlinear reduction is therefore important for recovering the fine-grained clusters this method targets: the

Cohort (principal question)	Statistic	Reduction method (to 5 dimensions) UMAP	PCA	t-SNE
French (feelings and sleep)	η^2	0.20 (24-27)	0.03 (2)	0.001 (2)
Italian (everyday life)	V	0.85 (4-6)	0.62 (2)	0.34 (2)
Chinese (neutral questions)	V	0.57 (3-6)	0.44 (3)	0.44 (3)
Spanish (sadness and coping)	V	0.27 (3-4)	0.16 (2)	0.26 (3)

Table S3. Effect size, with the number of nonnoise clusters in parentheses, under the published UMAP reduction and under PCA and t-SNE as standalone alternatives, each reducing the 4096-dimensional embeddings to the same 5 dimensions before identical HDBSCAN clustering, on the principal question of each cohort. The statistic is the Kruskal-Wallis η^2 for the continuous French score and Cramér V for the categorical clinical status. The UMAP column reproduces the five-dimensional setting of Table S2 (effect size averaged over 5 seeds, cluster count as the across-seed range); PCA is deterministic and t-SNE uses a fixed seed, so each alternative is a single run. UMAP gives the strongest association in every cohort and neither alternative improves on it.

choice of UMAP matters, whereas the number of components within that choice does not.

Internal cluster-validity indices

We complement the stability analysis with internal cluster-validity indices for the clustering. These indices are evaluated in the space the clustering is performed in, which here is the 5-dimensional UMAP space. We compute them on the nonnoise transcripts and report them across the same 10 random seeds as the stability table above (mean ± SD), so that each index is characterized by its seed variability. We report the density-based clustering validation index (DBCV) first, because it is designed for the density-based clusters HDBSCAN recovers and does not assume compact, convex groups (it ranges from -1 to 1, higher is better). We add the mean silhouette width (-1 to 1, higher is better) and the Davies-Bouldin index (0 upward, lower is better). All 3 use the Euclidean metric that HDBSCAN operates on in the reduced space.

Cohort (question)	Clusters	DBCV	Silhouette	Davies-Bouldin
French (feelings and sleep)	24-27	0.49 ± 0.05	0.61 ± 0.02	0.54 ± 0.03
Italian (everyday life)	4-6	0.61 ± 0.12	0.66 ± 0.07	0.49 ± 0.10
Chinese (neutral questions)	3-6	0.47 ± 0.06	0.50 ± 0.04	0.64 ± 0.06
Spanish (sadness and coping)	2-4	0.72 ± 0.10	0.73 ± 0.07	0.30 ± 0.09

Table S4. Internal cluster-validity indices in the 5-dimensional clustering space, across the same 10 random seeds as the stability table (mean ± SD), with HDBSCAN noise excluded. DBCV is the density-based clustering validation index (higher is better), the silhouette width is higher-is-better, and the Davies-Bouldin index is lower-is-better. The cluster-count ranges match the stability table above.

All 4 cohorts return a positive DBCV (0.47 to 0.72), a moderate-to-strong silhouette width (0.50 to 0.73), and a low Davies-Bouldin index (0.30 to 0.64), and all 3 are stable across seeds (small standard deviations), so the clusters are well separated in the space they are defined in. One caveat applies: these indices are computed in the same reduced space the clustering is performed in, so they are best read as a relative comparison across cohorts and alongside the results above, not as

standalone scores.

We also report a topic-coherence score, the standard word-co-occurrence metric for a topic model. Topic coherence asks whether the words that characterize a topic co-occur more often than chance, so it is computed on a word list rather than on a sentence. For each cluster we take its 10 most frequent content words, the conventional basis for topic coherence, and score their normalized pointwise mutual information (NPMI) over transcript-level co-occurrence, averaged over word pairs and over clusters (NPMI ranges from -1 to 1, higher is better). These most frequent words are not the keyword labels of the keyword baseline, which are the distinctive class-based TF-IDF terms, and not the language-model descriptions, which are sentences. Coherence is positive in every cohort (French 0.04, Italian 0.05, Chinese 0.09, Spanish 0.20), so each cluster's recurrent vocabulary co-occurs above chance. It is lowest for the French cohort, whose partition is the most fine-grained (26 clusters), since finer topics share more common vocabulary, which is the granularity the method targets. As with the separation indices, we read coherence as one descriptor among several rather than as a standalone verdict.

Bootstrap CIs for the reported effect sizes

The stability analysis above varies the clustering while holding the sample of participants fixed. A separate question is how much each effect size could have differed because we observed one finite sample rather than another comparable one. We answer it with a nonparametric percentile bootstrap. The 2 analyses are complementary and orthogonal. One varies the clustering and the other varies the sample, so an association is most credible when it is robust on both.

We drew 10,000 bootstrap resamples for every effect size stated in the manuscript, conditioning on the published partition (the same clustering used for every reported result, with HDBSCAN noise excluded). Conditioning on the partition means each transcript keeps its published cluster label: we do not rerun the clustering or reassign any transcript. Resampling the transcripts with replacement nonetheless changes how many of them fall in each cluster and which scores they carry, so each cluster's size and within-cluster score distribution vary from one resample to the next, even though the labels themselves never change. That variation is what moves the effect size. Each resample recomputes the same statistic on the resampled cluster-by-score pairs, Kruskal-Wallis eta-squared for a continuous score or Cramér V for a categorical status, and the 95% interval is the 2.5th to 97.5th percentile of the resampled values. Because the bootstrap conditions on the published partition, each point estimate equals the published value. That value is lower than the seed-averaged value in the stability table above, for the reason given above: reclustering reassigns points that the published run left as noise. For the French cohort the resampling unit is the participant rather than the transcript, because some participants contributed repeated visits. We resample participants with replacement and take all of their transcripts, matching the repeated-measures handling in Multimedia Appendix 2. In the 3 clinical cohorts each participant contributed a single transcript for the reported question, so a transcript-level resample is already a participant-level resample.

The manuscript reports the Chinese MDD association as a range across the 3 Chinese question groups (n=52). Their bootstrap 95% CIs are neutral questions 0.55 (0.36 to 0.78), positive questions 0.53 (0.36 to 0.76), and negative questions 0.40 (0.22 to 0.66), so the intervals span 0.22 to 0.78. Likewise, the abstract groups the French feelings-and-sleep associations for AIS, GAD-7, and MFI as eta-squared 0.14 to 0.16, whose intervals (above) span 0.12 to 0.21.

The French PHQ-9 and Italian depression associations are the most precisely estimated and are large

**Cohort (question, statistic)**	**n (clusters)**	**Resampling unit**	**Effect size (95% CI)**
French (feelings and sleep, PHQ-9 η^2)	1645 (26)	participant	0.19 (0.17 to 0.24)
Italian (everyday life, DSM-5 depression V)	106 (5)	transcript	0.74 (0.65 to 0.85)
Chinese (neutral questions, MDD V)	52 (4)	transcript	0.55 (0.36 to 0.78)
Spanish (sadness and coping, C-SSRS V)	58 (5)	transcript	0.50 (0.37 to 0.71)

Table S5. Bootstrap 95% CIs for the strongest effect size reported in each cohort. The n is the number of nonnoise transcripts entering the test, which is smaller than the n in the stability table above because that table counts all transcripts entering the clustering. The effect size is the Kruskal-Wallis eta-squared (French) or Cramér V (clinical cohorts). The Spanish value is the single published run and is treated as exploratory. Its seed-averaged value is Cramér V=0.27, reported above.

**Question**	**Score (statistic)**	**Effect size (95% CI)**
Current feelings and sleep	AIS η^2	0.15 (0.13 to 0.20)
Current feelings and sleep	BDI η^2	0.16 (0.14 to 0.21)
Current feelings and sleep	GAD-7 η^2	0.16 (0.14 to 0.21)
Current feelings and sleep	MFI η^2	0.14 (0.12 to 0.19)
Past negative event	AIS η^2	0.01 (0.01 to 0.04)
Past negative event	BDI η^2	0.01 (0.01 to 0.05)
Past negative event	GAD-7 η^2	0.02 (0.02 to 0.06)
Past negative event	MFI η^2	0.01 (0.01 to 0.05)
Past negative event	PHQ-9 η^2	0.03 (0.02 to 0.06)
Last 24 hours	age η^2	0.27 (0.24 to 0.32)
Last 24 hours	sex (Cramér V)	0.22 (0.21 to 0.30)

Table S6. Bootstrap 95% CIs for the secondary effect sizes stated in the manuscript body, all in the French general population cohort and resampled by participant. The French secondary tests run on the nonnoise transcripts of each question, from about 1356 to 1645 transcripts contributed by about 1200 to 1285 participants.

(0.19, 0.17 to 0.24; 0.74, 0.65 to 0.85). The Chinese MDD association is large in point estimate but far less precisely estimated: its interval is wide (0.55, 0.36 to 0.78) and spans the medium-to-large range, as expected at n=52. The Spanish interval is wider still (0.37 to 0.71) and, as the seed analysis above shows, unstable across clusterings, which is why we report the Spanish suicide-risk association as exploratory. All of these intervals are conditional on the reported partition, so they bound the sampling uncertainty for a fixed clustering rather than the additional variation from reclustering, which the seed analysis above characterizes separately. The 2 smaller clinical cohorts should therefore be read with the width of both in mind, and their precision should not be overstated. On the feelings-and-sleep question every French clinical score has a tight interval in the large range, while on the past-negative-event question every interval sits near zero. This separation between the associated and non-associated questions is therefore not an artifact of sampling.
