# Supplementary material for: Interpretable Topic Modeling of Spontaneous Speech in Depression Using Large Language Models: Multilingual Four-Cohort Study
Source: JMIR Ment Health. 2026 Aug 17;13:e99185. doi: 10.2196/99185 (PMC13480703; doi:10.2196/99185)
Supplement: Supplementary file 5 [file mental-v13-e99185-s005.pdf]

Pipeline hyperparameters and the cluster-description prompt

This appendix gives the hyperparameters for each step of the computational pipeline and the exact prompts used for text embedding and cluster description. It supports the manuscript "Interpretable Topic Modeling of Spontaneous Speech in Depression Using Large Language Models: A Multilingual Four-Cohort Study."

Text embedding

To embed transcripts into 4096-dimensional vectors, we used Qwen3-Embedding-8B (model weights: Qwen/Qwen3-Embedding-8B on Hugging Face), an 8 billion parameter language model trained for generating text embeddings suitable for various tasks such as retrieval or clustering. We used a context length of 2048 tokens, and the following instruction:

```
Identify the topic or theme of the given transcript
```

Dimension reduction

We used UMAP to reduce the 4096-dimensional embeddings to the 5 dimensions on which HDBSCAN clusters, with 30 neighbors, a cosine metric, and a minimum distance of 0 so that points in the same neighborhood are packed densely and HDBSCAN can recover dense regions. These values follow the configuration recommended by the library maintainers for the downstream-clustering use case [1]. The dimension reduction step facilitates density-based clustering, which otherwise suffers from the curse of dimensionality.

Clustering

We used a minimum cluster size of 30 transcripts. A fixed floor of 30 is too large for small cohorts: with 52 transcripts, the Chinese cohort (MODMA) would yield at most 1 cluster, so for such cohorts we set the minimum cluster size to one-tenth of the cohort. Multimedia Appendix 4 sweeps this minimum from half to twice its value and finds the reported associations stable to the choice.

Cluster labeling

We used Qwen3-14B (model weights: Qwen/Qwen3-14B on Hugging Face), a 14 billion parameter language model, with a context length of 40960 tokens, and the following instruction to summarize 30 random transcripts taken from a cluster:

```
Give a summary of the transcripts from multiple individuals.
Here is the transcripts:\n\n{transcripts}. Answer in English.
```

```
The summary must indicate the common themes between individuals.
The themes must be specific, do not be too general, and do not
say obvious things such as 'reflect on their emotions, past
decisions and experiences', 'share experiences of both positive
and negative events', 'dealing with challenges', 'discuss their
recent activities and future plans'.\n\nNow, give the summary
of the texts in one sentence, starting with 'The individuals express'
```

We used the default generation hyperparameters recommended by the maintainers of Qwen3: temperature of 0.6, top-k of 20, and top-p of 0.95 with the thinking mode enabled.

Clinically agnostic prompt

We deliberately used a single, clinically agnostic prompt that asks only for the common themes among transcripts, with no reference to symptoms, diagnoses, or any construct we later test for. Injecting clinically loaded prompts (for example, asking the model to describe "depressive symptoms") would steer descriptions toward the very constructs against which we evaluate cluster membership, reintroducing the circularity the design avoids. The descriptions are therefore generated independently of the clinical labels, and their association with clinical scores is established by the statistical tests, not by the prompt. The relevant robustness questions for this design are stochastic stability (sampling and seed) and robustness to the wording of the prompt, both of which we address in Multimedia Appendix 6, where the descriptions prove stable to neutral paraphrases of the prompt. For a dedicated clinical application, the same pipeline can use a domain-specific prompt instead, provided the prompt does not reference the constructs the descriptions are later tested against, which would otherwise reintroduce the circularity this design avoids.

References

[1] Leland McInnes, John Healy, and James Melville. UMAP: Uniform Manifold Approximation and Projection for Dimension Reduction, September 2020. URL http://arxiv.org/abs/1802.03426. arXiv:1802.03426 [stat].
