# Supplementary material for: Interpretable Topic Modeling of Spontaneous Speech in Depression Using Large Language Models: Multilingual Four-Cohort Study
Source: JMIR Ment Health. 2026 Aug 17;13:e99185. doi: 10.2196/99185 (PMC13480703; doi:10.2196/99185)
Supplement: Supplementary file 6 [file mental-v13-e99185-s006.pdf]

Stability of the language-model cluster descriptions across transcript sampling, generation, and prompt wording

This appendix quantifies how reproducible the language-model cluster descriptions are when the description-generation step is repeated, across the random sampling of transcripts, the stochastic generation of the language model, and the wording of the prompt, for every interview question analyzed in each cohort. It supports the manuscript "Interpretable Topic Modeling of Spontaneous Speech in Depression Using Large Language Models: A Multilingual Four-Cohort Study" and complements the clustering-stability analysis in Multimedia Appendix 4: that appendix fixes the descriptions and varies the clustering, whereas here we fix the clustering and vary the descriptions.

Purpose

The cluster descriptions are the interpretability layer of the pipeline. Each description is produced by sampling 30 of a cluster's transcripts and asking a language model to summarize them under a single clinically agnostic prompt (Multimedia Appendix 5). We quantify how much the descriptions vary when that step is repeated, so that the interpretive weight the manuscript places on them is matched by direct evidence of their reproducibility. Every reported statistical association is computed from cluster membership, not from the descriptions, so this analysis concerns only the interpretability layer and leaves all quantitative findings unchanged.

Method

We held the published clustering fixed and reran only the description-generation step, for every cluster of every analyzed interview question in each cohort (the same 13 cohort-questions as Multimedia Appendix 4). We did not recluster, so the partitions are exactly those that underlie every manuscript figure and reported description. For each cluster we regenerated the description 20 times.

Two sources of variation distinguish one regeneration from the next. The first is the transcript sample: each regeneration draws a fresh random sample of 30 transcripts from the cluster, or uses the whole cluster when it holds fewer than 30. The second is the language model's generation, which is stochastic rather than deterministic: the model produces text by sampling at the generation temperature reported in Multimedia Appendix 5, so it does not return an identical summary on repeated runs even from the same input. Each regeneration uses a fresh random seed for both the sample and the model, and otherwise repeats the manuscript procedure exactly, asking for a one-sentence summary under the same clinically agnostic prompt.

The cluster sizes determine which of the 2 sources is active. In the French general-population cohort the clusters hold 30 to 114 transcripts, so the 30-transcript sample genuinely varies from run to run. In the smaller clinical cohorts every cluster holds at most 30 transcripts, so the whole cluster enters every run and only the model's generation varies. The French cohort therefore probes transcript sampling and generation jointly, and the clinical cohorts probe generation alone.

We separately tested robustness to the wording of the prompt. Holding the clustering and a fixed 30-transcript sample per cluster constant, we regenerated each cluster's description under 3 phrasings of the prompt: the published prompt (Multimedia Appendix 5) and the 2 neutral paraphrases below. Each phrasing asks only for the common themes among the transcripts and names no symptom, diagnosis, or construct we later test, so this isolates the effect of wording. Because the sample is held fixed, the only thing that varies across the three is the prompt.

```
Summarize what these transcripts from different individuals
have in common:\n\n{transcripts}\n\nWrite one English sentence
beginning 'The individuals express'. Name concrete, specific
shared themes and avoid generic statements.
```

```
Across the following transcripts from several people, identify
the themes they share:\n\n{transcripts}\n\nRespond in English
with a single specific sentence starting 'The individuals
express', naming concrete themes rather than broad generalities.
```

We embedded every regenerated description with the study's embedding model (Qwen3-Embedding-8B; Multimedia Appendix 5) and report 3 quantities. The within-cluster similarity is the mean pairwise cosine similarity of a cluster's regenerations, where 1 denotes identical meaning; this is the primary measure of reproducibility. The agreement with the reported description is the cosine similarity of the regenerations to the description printed in the manuscript. The word overlap is the mean proportion of shared content words, which credits literal word reuse only and ignores paraphrase. As a reference baseline, we also compute the similarity between descriptions of different clusters.

Results

Across the 161 clusters of the 13 cohort-questions (20 regenerations each, 3220 descriptions in total), the regenerated descriptions of a cluster are highly consistent. The within-cluster similarity is 0.84 to 0.92 per cohort-question, with a per-cluster median of 0.91, and 88% of clusters are at or above 0.85. The regenerations also stay close to the descriptions reported in the manuscript (mean similarity 0.82). The word overlap is low throughout (0.19 to 0.25), so the run-to-run variation is paraphrase rather than a change of theme. Because every description is a one-sentence summary of the same kind of interview material, these embedding cosines are high in absolute terms even for unrelated descriptions: the mean similarity between descriptions of different clusters is 0.71. What matters is the consistent gap above that level, as every cohort-question's within-cluster similarity (0.84 to 0.92) sits above it. Read together with the low word overlap, the high within-cluster similarity therefore reflects genuine agreement on theme rather than all descriptions merely resembling one another.

To make this concrete, the manuscript's reported description for one French feelings-and-sleep cluster (the academic-stress cluster, within-cluster similarity 0.93) is shown below with one of its regenerations. Both name academic and exam pressure, sleep disruption, and partial relief through routine or social support, and differ only in wording and level of detail.

**Question**	**Clusters (n)**	**Within-cluster similarity (mean ± SD)**	**Agreement with reported description**	**Word overlap**
French general population (clusters of 30-114 transcripts: sampling and generation both vary)				
Last 24 hours	25	0.88 ± 0.04	0.82	0.23
Past negative event	18	0.89 ± 0.04	0.84	0.21
Past positive event	20	0.92 ± 0.03	0.86	0.24
Future negative event	19	0.91 ± 0.03	0.89	0.23
Future positive event	23	0.92 ± 0.03	0.89	0.25
Current feelings and sleep	26	0.92 ± 0.02	0.89	0.23
Italian clinical, Androids (clusters of at most 30 transcripts: generation only)				
Everyday life	5	0.88 ± 0.03	0.81	0.21
Chinese clinical, MODMA (clusters of at most 30 transcripts: generation only)				
Neutral questions	4	0.88 ± 0.01	0.83	0.22
Positive questions	5	0.86 ± 0.02	0.68	0.24
Negative questions	4	0.87 ± 0.02	0.82	0.21
Spanish clinical, VOCES (clusters of at most 30 transcripts: generation only)				
Sadness and coping	5	0.91 ± 0.02	0.82	0.22
Average day	4	0.85 ± 0.06	0.70	0.21
Recent good news	3	0.84 ± 0.05	0.77	0.19

Table S1. Stability of the cluster descriptions across 20 regenerations per cluster, for every interview question analyzed in each cohort. Each regeneration re-draws the 30-transcript sample and reruns the language model with a fresh seed. Within-cluster similarity is the mean pairwise cosine similarity of a cluster's 20 regenerated descriptions, embedded with Qwen3-Embedding-8B, averaged over clusters (mean ± SD across clusters). Agreement with the reported description is the mean cosine similarity of the regenerations to the description printed in the manuscript. Word overlap is the mean pairwise proportion of shared content words. The number of clusters is the count for each question.

Reported. *The individuals express stress and anxiety related to academic pressures, internships, and future exams, alongside varied sleep patterns, including difficulty falling asleep, early awakenings, and efforts to maintain restful sleep schedules, while some note improvements in well-being through social support, transitions in education, and temporary relief after completing key milestones.*

Regeneration. *The individuals express a recurring pattern of sleep disruption tied to academic pressure, particularly during exam periods, characterized by delayed sleep onset due to rumination, anxiety over results and future uncertainty, and fragmented or insufficient sleep, while some report improved sleep post-exams or during breaks, and a few describe stable or good sleep despite ongoing stress.*

Rewording the prompt leaves the descriptions equally stable. Across the 3 phrasings, a cluster's descriptions reach a mean pairwise similarity of 0.88, and they stay close to the manuscript description (mean similarity 0.78), comparable to the sampling-and-generation stability above. Rewording the clinically agnostic prompt therefore restates the same themes rather than changing them.

**Cohort (question)**	**Clusters (n)**	**Cross-prompt similarity**	**Agreement with reported description**
French (feelings and sleep)	26	0.89	0.81
Italian (everyday life)	5	0.84	0.68
Chinese (neutral questions)	4	0.85	0.75
Spanish (sadness and coping)	5	0.88	0.77

Table S2. Stability of the cluster descriptions across 3 phrasings of the clinically agnostic prompt (the published prompt and 2 neutral paraphrases of it; Multimedia Appendix 5), with the transcript sample held fixed per cluster. Cross-prompt similarity is the mean pairwise cosine of a cluster's 3 descriptions, embedded with Qwen3-Embedding-8B, averaged over clusters. Agreement with the reported description is the mean cosine similarity of those 3 descriptions to the description printed in the manuscript, again averaged over clusters.

Interpretation

The regenerated descriptions are reproducible. The within-cluster similarity is uniformly high, with per-cohort means of 0.84 to 0.92 and a per-cluster median of 0.91, so rerunning the description step recovers the same content rather than a description that drifts from cluster to cluster.

The 2 sources of variation behave as expected. Stability is comparable whether the transcript sample is re-drawn each time (French, 0.88 to 0.92) or held fixed so that only the model's generation varies (clinical cohorts, 0.84 to 0.91), so resampling which transcripts are shown does not destabilize the descriptions. The variation that remains is paraphrase: the word overlap is low while the similarity is high, so regenerations restate the same themes in different words, as the qualitative example shows. The regenerations also stay close to the descriptions printed in the manuscript (mean similarity 0.82, range 0.68 to 0.89), so those descriptions are representative runs rather than unusually favorable ones.

The least stable individual clusters, near a within-cluster similarity of 0.78, are the small clinical clusters and the largest, most varied French clusters. These are the cases where a single description

should be read with the most caution. Rewording the prompt is tolerated equally well (cross-phrasing similarity 0.88), so the descriptions are stable to neutral rewordings of the prompt as well as to transcript sampling and generation. Prompts that name a target symptom, diagnosis, or construct are deliberately out of scope: by design they would steer the descriptions toward the very clinical constructs these tests evaluate, reintroducing the circularity the clinically agnostic prompt avoids (Multimedia Appendix 5). This analysis therefore bounds sensitivity to wording within the clinically agnostic regime, not across prompt intent.

This analysis concerns only the interpretability layer, and it bounds run-to-run variation rather than correctness. Reproducibility means the description step recovers the same content when repeated. It is not evidence that a description is clinically accurate. Every statistical association in the manuscript is computed from cluster membership, not from the descriptions, so these results quantify the reproducibility of the descriptions without affecting any quantitative finding. They also support the manuscript's statement that the 30-transcript sample produces stable descriptions across runs.
