# Supplementary material for: Interpretable Topic Modeling of Spontaneous Speech in Depression Using Large Language Models: Multilingual Four-Cohort Study
Source: JMIR Ment Health. 2026 Aug 17;13:e99185. doi: 10.2196/99185 (PMC13480703; doi:10.2196/99185)
Supplement: Supplementary file 7 [file mental-v13-e99185-s007.pdf]

Small-cohort Monte-Carlo permutation tests with expected cell counts

This appendix reports expected cell counts and assumption-free Monte-Carlo permutation tests for the small clinical cohorts. It supports the manuscript "Interpretable Topic Modeling of Spontaneous Speech in Depression Using Large Language Models: A Multilingual Four-Cohort Study."

Purpose

With small clinical cohorts and several clusters, the Pearson chi-square test of cluster membership by the diagnostic or risk group (the binary clinical grouping of each cohort, for example depression status, MDD diagnosis, or suicide-risk class) can have sparse expected cell counts, which violates its large-sample assumption and can distort the asymptotic P value. We therefore report the expected cell counts and recompute each principal association with an assumption-free Monte-Carlo permutation chi-square test. The French general population cohort is not included, because its large sample produces no sparse expected cells and the asymptotic test needs no such check.

Method

For each small clinical cohort we built the contingency table of cluster membership by the diagnostic or risk group from the primary clustering (the same clustering used for every result reported in the study), excluding noise points, recomputed the Pearson chi-square statistic and Cramér V, and reported the minimum expected cell count and the percentage of cells with expected count below 5. We then computed a Monte-Carlo permutation P value by shuffling the group labels against the fixed cluster labels (20,000 permutations), which preserves both margins and requires no large-sample assumption. This test therefore asks a single question, whether the association in the reported contingency table is robust to sparse expected counts. It is independent of the separate stability analysis (Multimedia Appendix 4), which asks whether the association persists when the clustering is recomputed across random seeds.

Results

Interpretation

The chi-square assumptions are largely met: in the Italian cohort no cell had an expected count below 5, and in the Chinese and Spanish cohorts only 12% and 20% of cells did, with minimum expected counts near 4. Where cells were sparse, the assumption-free Monte-Carlo permutation test agreed with the asymptotic test, and all 3 associations remained significant (P<.01). For the Italian cohort the Monte-Carlo P reached the resolution floor of a 20,000-permutation test (P<.001, the smallest value such a test can return, 1/20,000): no permuted table reached the observed association,

Cohort	Status	Table	n	Cramér V	Min expected	Cells <5	Asymptotic P	Monte-Carlo P
Italian (Androids)	DSM-5 depression	5×2	106	0.74	6.13	0%	<.001	<.001
Chinese (MODMA)	MDD diagnosis	4×2	52	0.55	4.42	12%	.001	.001
Spanish (VOCES)	C-SSRS risk class	5×2	58	0.50	3.88	20%	.006	.005

Table S1. Small-cohort chi-square diagnostics and assumption-free Monte-Carlo permutation tests. The Cramér V shown is the value from the primary (reported) clustering. "Min expected" is the minimum expected cell count and "Cells <5" the percentage of cells with expected count below 5. The Monte-Carlo P value is from 20,000 label permutations preserving both margins. The reported n is the number of nonnoise transcripts entering each test.

so the permutation test confirms the asymptotic result up to its own resolution. The reported clinical associations are therefore not an artifact of small expected counts.

For the Spanish cohort, the Cramér V of 0.50 above comes from the single primary clustering run, and this appendix uses it only to confirm that its P value is not inflated by sparse cells. This single-run value is not the effect size the study reports for the cohort. Because the Spanish suicide-risk association varies with the clustering seed (Multimedia Appendix 4), the manuscript reports the seed-averaged Cramér V=0.27 and labels the finding exploratory. For the Italian and Chinese cohorts the association is stable across seeds, so the single-run value above matches the value the manuscript reports.
