# Supplementary material for: Interpretable Topic Modeling of Spontaneous Speech in Depression Using Large Language Models: Multilingual Four-Cohort Study
Source: JMIR Ment Health. 2026 Aug 17;13:e99185. doi: 10.2196/99185 (PMC13480703; doi:10.2196/99185)
Supplement: Supplementary file 8 [file mental-v13-e99185-s008.pdf]

Keyword labels vs language-model descriptions

This appendix compares keyword labels with language-model descriptions for the same clusters. It supports the manuscript "Interpretable Topic Modeling of Spontaneous Speech in Depression Using Large Language Models: A Multilingual Four-Cohort Study."

Purpose

Keyword-based topic models are the standard baseline for computational topic modeling. Established methods such as Latent Dirichlet Allocation [1] and BERTopic [2] label each cluster with a ranked list of characteristic words. This word-list representation is the conventional, most widely used way to describe a topic. It is therefore the natural baseline for our method, which keeps the same clustering but replaces the word list with a language-model description of the cluster. This appendix makes the comparison concrete by labeling the same clusters both ways. Both label types describe the same clusters, built from transcripts only and specific to a cohort and task, so the clusters and every statistical test are identical regardless of how the clusters are labeled. The comparison is therefore purely about what a reader can take from the label.

Method

For each cluster we produced 2 labels. The keyword label is the standard output of a keyword-based topic model (BERTopic): its class-based term frequency-inverse document frequency (c-TF-IDF), which down-weights words common across clusters, computed over the fixed cluster assignments after removing common stopwords in each language, keeping the 10 highest-weighted terms, translated into English below (the originals are French, Italian, Chinese, and Spanish). The description label is the language-model summary already generated in the pipeline, and is reproduced verbatim below, exactly as the model returned it, without any editing or shortening. We report this comparison qualitatively and side by side rather than through an automatic readability score, because the property at issue, interpretability, is a judgment made by a human reader. A topic-coherence score does not measure readability here. Topic coherence scores a word list by how often its words co-occur, so it applies to the keyword label but not to a language-model description, which is a sentence rather than a word list, and so cannot compare the two. Even on the keyword label it would reward the distinctive, cluster-specific words that c-TF-IDF surfaces, the same idiosyncratic tokens that make a keyword list hard to read, rather than the readability that separates the 2 label types. The topic coherence of the clusters themselves, computed on their most frequent words, is reported as a cluster-quality index in Multimedia Appendix 4. A side-by-side presentation lets the reader weigh the 2 label types directly, the appropriate standard of evidence for a claim about readability.

The first column of each table is a short name we assigned to each cluster for ease of reference only. These names are not produced by either method and play no role in the analysis. The 2 automatically generated labels that the comparison concerns are the keyword label and the language-model description.

Examples

We show a representative selection of clusters for each cohort rather than every cluster. The French cohort alone has about 26 clusters, so an exhaustive listing would be unwieldy, and the contrast described below recurs across clusters rather than depending on any particular one. For each cohort the selection spans clusters of differing clinical character, including the most and least symptomatic in the clinical cohorts, so it illustrates the comparison without being chosen to favor either label type.

French general population, current feelings and sleep (PHQ-9)

Cluster	Keyword label (translated)	Language-model description (verbatim)
Academic stress (young adults)	exams, midterms, classes, stressed, competitive exams, semester, studies	The individuals express stress and anxiety related to academic pressures, internships, and future exams, alongside varied sleep patterns, including difficulty falling asleep, early awakenings, and efforts to maintain restful sleep schedules, while some note improvements in well-being through social support, transitions in education, and temporary relief after completing key milestones.
Restful sleep and well-being	perfectly, pleasant, gentle, restful, short (nights)	The individuals express consistent satisfaction with their current well-being, emphasizing good sleep quality, restful or pleasant nights, and a general sense of relaxation, even when noting variations in sleep duration or occasional fatigue.
Health and illness	covid, operation, health, illness, sick, fatigue, doctor, cold	The individuals express a mix of physical health challenges such as chronic pain, fatigue, and medical conditions (e.g., endometriosis, post-operative recovery, respiratory infections) impacting their sleep quality and energy levels, alongside a persistent emphasis on maintaining a positive outlook, adapting to daily limitations, and managing symptoms through rest, lifestyle adjustments, or medical treatment.
Pregnancy and childcare	children, husband, baby, menopause, pregnant, pregnancy, daughter, home	The individuals express challenges related to disrupted sleep due to pregnancy, childcare responsibilities, and work demands, alongside emotional strain from relationship dynamics and the stress of life transitions such as preparing for a new baby or relocating.

Italian clinical, Androids, everyday life (DSM-5 depression)

Cluster	Keyword label (translated)	Language-model description (verbatim)
Most depressed	home, period, depression, work, mother, family	The individuals express persistent struggles with depression, anxiety, and panic attacks, often exacerbated by significant life events such as family responsibilities, health-related stress from routine medical check-ups, and a pervasive sense of stagnation and purposelessness amid repetitive daily routines, while some also grapple with disrupted academic or career goals and complex family dynamics.
Least depressed	likes, studying, family, Naples, parents, singing	The individuals express a shared focus on family dynamics and intergenerational relationships, regional identity and emotional ties to Naples, and the pursuit of personal passions such as music, research, and education, while reflecting on the complexities of balancing domestic responsibilities with professional or creative endeavors.
Marital and domestic conflict	husband, mother, things, person, photo, Riccardo (name), girls	The individuals express challenges related to domestic and marital conflicts, feelings of isolation and emotional exhaustion, the struggle to navigate complex family dynamics and expectations, and the interplay between personal resilience and the need for social or familial support.

Chinese clinical, MODMA, neutral questions (MDD diagnosis)

Cluster	Keyword label (translated)	Language-model description (verbatim)
Most depressed	feeling, especially, think, before, now, nothing, seem, do not want, life	The individuals express how physical health issues and emotional struggles such as depression, anxiety, and loss of motivation significantly impact their daily lives, relationships, and work, while also highlighting the complex role of supportive friends and family, as well as persistent feelings of inadequacy and communication challenges within familial contexts.
Personal struggles	time, before, work, heating, son, not good, school, likes, talking	The individuals express a range of personal struggles including sleep disturbances, lack of confidence, difficulty in maintaining social relationships, physical health issues such as headaches and stomach problems, and challenges in emotional regulation, while also mentioning some positive aspects like supportive friends, hobbies such as photography and sports, and aspirations related to personal growth and family.

Spanish clinical, VOCES, sadness and coping (C-SSRS risk class)

Cluster	Keyword label (translated)	Language-model description (verbatim)
Sadness coping	sad, bad, to feel, sensation, going out, music, week, news, Madrid	The individuals express diverse coping mechanisms such as utilizing music and creative outlets, engaging in physical activities like exercise or outdoor pursuits, seeking social support through communication, employing distraction via academic or intellectual tasks, and occasionally resorting to maladaptive strategies like self-inflicted physical pain, while some emphasize adaptability in modifying plans and self-reflection as part of their emotional management.
Low motivation / apathy	minutes, unmotivated, want, activity, low mood, apathetic, desire	The individuals express common strategies for coping with low moods, including using media (e.g., TV, YouTube, music), engaging in physical activities (e.g., walking, cleaning), seeking social support (e.g., talking to friends, family), and employing distraction techniques (e.g., gaming, napping) to avoid rumination, while some emphasize the importance of acceptance, structured routines, and medication in managing emotional distress.

Interpretation

The same 3 differences recur across cohorts and languages. (1) Keyword lists mix the central theme with idiosyncratic or generic words that are locally distinctive to a cluster, such as proper names (Naples, Madrid, Riccardo). For the most depressed Chinese cluster they are almost only generic words (*feeling*, *think*, *now*, *do not want*) that convey no clinical content, which the reader must work around. (2) Keyword lists are unordered and leave the clinical character implicit, whereas the descriptions name it. The most depressed Italian and Chinese clusters yield descriptions stating *depression, anxiety, and panic attacks* and *depression, anxiety, and loss of motivation*, content a reader could not reliably infer from the word lists alone. (3) The descriptions also surface clinically important content a word list omits, and they draw protective-versus-risk distinctions. The Spanish coping cluster's description reports *self-inflicted physical pain* as a maladaptive strategy that does not appear in its keywords. Elsewhere they separate *restful or pleasant nights* from *difficulty falling asleep, early awakenings*, where a bare keyword such as *sleep* would not. These differences are the interpretability advantage the study claims for the descriptions. The advantage concerns readability and clinical legibility for a human reader, and it leaves every quantitative result unchanged, because both label types describe the same clusters.

References

[1] David M. Blei, Andrew Y. Ng, and Michael I. Jordan. Latent Dirichlet Allocation. *Journal of Machine Learning Research*, 3(Jan):993–1022, 2003. ISSN ISSN 1533-7928. URL `https://jmlr.csail.mit.edu/papers/v3/blei03a.html`.

[2] Maarten Grootendorst. BERTopic: Neural topic modeling with a class-based TF-IDF procedure, March 2022. URL `http://arxiv.org/abs/2203.05794`. arXiv:2203.05794 [cs].
