# Supplementary material for: Interpretable Topic Modeling of Spontaneous Speech in Depression Using Large Language Models: Multilingual Four-Cohort Study
Source: JMIR Ment Health. 2026 Aug 17;13:e99185. doi: 10.2196/99185 (PMC13480703; doi:10.2196/99185)
Supplement: Supplementary file 9 [file mental-v13-e99185-s009.pdf]

Faithfulness of the cluster descriptions to their transcripts

This appendix examines whether each language-model cluster description asserts content that the transcripts it was generated from do not support. It supports the manuscript "Interpretable Topic Modeling of Spontaneous Speech in Depression Using Large Language Models: A Multilingual Four-Cohort Study" and complements Multimedia Appendix 6. Multimedia Appendix 6 asks whether the descriptions are reproducible when the description step is repeated. This appendix asks whether they are faithful to the transcripts they summarize.

Purpose

The descriptions are the interpretability layer of the pipeline. A reader should be able to trust that each one reflects its transcripts rather than introducing content they do not contain. We therefore examined every cluster description of the 4 interview questions the manuscript presents in its main figures: the French feelings-and-sleep question, the Italian everyday-life interview, the Chinese neutral questions, and the Spanish sadness-and-coping question. These 4 questions yield 40 clusters in total. We judged whether each description is faithful to those transcripts or asserts content they do not support. Every statistical association in the manuscript is computed from cluster membership, not from the descriptions. This analysis therefore concerns how far the interpretability layer can be trusted, and does not affect any quantitative finding.

Method

Each description is a one-sentence summary the language model produced from 30 transcripts sampled at random from the cluster, or from the whole cluster when it holds 30 or fewer (manuscript Methods and Multimedia Appendix 5). We examined every description against exactly those transcripts, the ones the model was shown when it generated the description. This makes the analysis a direct test of the generation step. It asks whether the model's summary stays within what it was given. Every Italian, Chinese, and Spanish cluster holds at most 30 transcripts, and so do the smaller French clusters. In those cases the examined transcripts are the entire cluster. Only the larger French clusters were summarized from a 30-transcript sample.

We read each description against its transcripts and checked every theme it names. Whether a description asserts content its transcripts do not support is a matter of comparing the summary with the source text rather than a clinical judgment, and is distinct from evaluating the descriptions' clinical accuracy, completeness, or relevance. We classified a description as faithful when the transcripts support every theme, and unfaithful when it asserts a theme or an organizing structure the transcripts do not support. Within the faithful descriptions, we also noted any case where a detail that appears in only a single transcript is stated without qualification, as if it were a general pattern. This overstates the detail's prevalence, even though the content is genuinely present. A selection of these descriptions is reproduced verbatim in Multimedia Appendix 8.

Results

Of the 40 cluster descriptions, 39 were faithful to their transcripts and 1 was unfaithful (Table S1).

The unfaithful description is in the Spanish sadness-and-coping cohort. It frames the cluster as *two contrasting individuals*, one defined by *advanced digital literacy and routine use of technology* and the other by mental-health struggle. In fact the cluster is a set of 13 transcripts, the majority of which express sadness and recurrent suicidal ideation. The *digital literacy* content is not a participant's account of technology. It is the text of an interviewer-administered digital-skills questionnaire recorded in 3 of the transcripts. The description therefore imposes a structure the cluster does not have and treats that standardized-assessment text as a theme. This cluster belongs to the Spanish suicide-risk question, whose clinical association the manuscript already reports as exploratory and unstable across clustering seeds (Multimedia Appendix 4).

Three of the 39 faithful descriptions state a single-transcript detail without qualifying it as exceptional: in the French feelings-and-sleep cohort, *self-esteem issues* in one high-symptom cluster and *reliance on sleep aids like noise* in another, and in the Italian everyday-life cohort, *work in retail or sales* in the daily-routines cluster. In each case the detail is genuinely present in the cluster. Only its prevalence is overstated, and no content is introduced that the transcripts do not contain.

**Cohort**	**Descriptions (n)**	**Faithful**	**Unfaithful**
French (feelings and sleep)	26	26	0
Italian (everyday life)	5	5	0
Chinese (neutral questions)	4	4	0
Spanish (sadness and coping)	5	4	1
Overall	40	39	1

Table S1. Faithfulness of the cluster descriptions to the transcripts they were generated from, for the 4 interview questions analyzed in the manuscript (40 clusters). A description is faithful when the transcripts support every theme it names and unfaithful when it asserts a theme or organizing structure the transcripts do not support. The unfaithful description (Spanish sadness-and-coping) imposes a *two contrasting individuals* structure the cluster lacks and treats interviewer-administered digital-skills questionnaire text as a theme. Three of the 39 faithful descriptions, 2 French and 1 Italian, additionally state a single-transcript detail without qualification. This overstates the detail's prevalence but introduces no content absent from the cluster.

Interpretation

The descriptions are faithful to the transcripts they summarize. Of the 40, 39 assert only content the transcripts support. The one exception is a structural misreading rather than invented content, traceable to interviewer-administered questionnaire text that entered a few transcripts. In the few descriptions that fall short of full faithfulness, the error is to overstate how common a detail is, not to introduce a detail the transcripts do not contain. Because each description was examined against the very transcripts the model was shown, any departure is attributable to the generation step itself. For the clinical cohorts and the smaller French clusters, where the model saw the whole cluster, the check covers every transcript. The manuscript relates these descriptions to the risk and protective topics established in psychiatric research (manuscript, Risk and protective topics for depression). Because

the descriptions stay within their transcripts, that relation rests on content the speakers expressed, not content the model introduced. Every statistical association in the manuscript is computed from cluster membership, not from the descriptions, so these findings concern the interpretability layer alone and leave every quantitative result unchanged.
