# Supplementary material for: Interpretable Topic Modeling of Spontaneous Speech in Depression Using Large Language Models: Multilingual Four-Cohort Study
Source: JMIR Ment Health. 2026 Aug 17;13:e99185. doi: 10.2196/99185 (PMC13480703; doi:10.2196/99185)
Supplement: Supplementary file 10 [file mental-v13-e99185-s010.pdf]

Computational cost and comparison with manual thematic analysis

This appendix estimates the analyst time that a manual thematic analysis of one interview question would take and sets it against the measured running time and cost of the automated pipeline. It supports the manuscript "Interpretable Topic Modeling of Spontaneous Speech in Depression Using Large Language Models: A Multilingual Four-Cohort Study" and the Discussion subsection "Bridging qualitative research and clinical practice."

Purpose

Manual thematic analysis is clinically valuable but does not scale to large corpora. This comparison is illustrative: it contrasts the scale of the 2 workflows and is not a claim that the automated pipeline reproduces a manual thematic analysis. The pipeline is an automated topic-modeling procedure (embedding, clustering, and language-model description) that accelerates the 3 early stages of thematic analysis, familiarization with the material, initial coding, and theme generation [1], while the interpretive coding, validation, and clinical interpretation of the themes remain with the clinician. The comparison covers only those 3 stages, since the rest is shared by both workflows, and excludes transcription, since both begin from the same transcripts. The manual times are estimates from the corpus size (Multimedia Appendix 1) and published analyst rates.

Method

We estimate the manual effort for the French feelings-and-sleep question ("Describe how you are feeling at the moment and how your sleep has been lately"), the question the manuscript analyzes in detail (about 148,000 words; Multimedia Appendix 1). For familiarization we assume the analyst reads the transcripts at 238 words per minute, the average adult silent-reading rate [2]. For initial coding we assume 2 to 4 minutes per transcript, a conservative rate for short open-ended answers [3]. Searching for and defining themes from the codes is a further phase [1], and reliable analysis requires independent double-coding by at least 2 coders with reconciliation of disagreements [4].

We measured the pipeline's running time on a single NVIDIA A100 GPU (64 GB), using the configuration described in the Methods and Multimedia Appendix 5. Cost is the on-demand cloud rental of an equivalent A100 GPU, at $1.40 to $2.80 per GPU-hour [5, 6].

Results

Reading the transcripts takes about 10 hours and initial coding 60 to 120 hours. With the further work of searching for and defining themes, a single-coder analysis comes to an estimated 90 to 170 hours, and roughly 180 to 340 hours under double-coding. This estimate is consistent with the 126.5 hours of researcher time measured for a full thematic analysis of a comparable qualitative dataset

[7]. Analyzing all 13 interview questions across the 4 cohorts by hand would run to several hundred hours.

The pipeline processes the same question in 167 seconds: embedding the transcripts in 31 seconds, reducing and clustering them in 17 seconds, and generating the cluster descriptions in 119 seconds. At the rental rates above, that question costs $0.06 to $0.13, and the 13 questions across the 4 cohorts together take 21 minutes of compute, $0.50 to $1.00.

Interpretation

Manual thematic analysis does not scale to thousands of transcripts across 4 cohorts, whereas the pipeline completes the same early stages in minutes. This illustrates the scale gap the pipeline addresses. It automates the early stages, not the interpretive coding and clinical judgment, which remain with the clinician.

References

[1] Virginia Braun and Victoria Clarke. Using thematic analysis in psychology. *Qualitative Research in Psychology*, 3(2):77–101, January 2006. ISSN 1478-0887, 1478-0895. doi: 10.1191/1478088706qp063oa. URL `http://www.tandfonline.com/doi/abs/10.1191/1478088706qp063oa`. Publisher: Informa UK Limited.

[2] Marc Brysbaert. How many words do we read per minute? A review and meta-analysis of reading rate. *Journal of Memory and Language*, 109:104047, 2019. doi: 10.1016/j.jml.2019.104047.

[3] Maximo R. Prescott, Samantha Yeager, Lillian Ham, Carlos D. Rivera Saldana, Vanessa Serrano, Joey Narez, Dafna Paltin, Jorge Delgado, David J. Moore, and Jessica Montoya. Comparing the Efficacy and Efficiency of Human and Generative AI: Qualitative Thematic Analyses. *JMIR AI*, 3:e54482, 2024. doi: 10.2196/54482.

[4] Cliodhna O'Connor and Helene Joffe. Intercoder Reliability in Qualitative Research: Debates and Practical Guidelines. *International Journal of Qualitative Methods*, 19:1609406919899220, 2020. doi: 10.1177/1609406919899220.

[5] Lambda. On-demand GPU cloud pricing. `https://lambda.ai/pricing`, 2026. accessed June 2026.

[6] GetDeploying. NVIDIA A100 cloud GPU pricing across providers. `https://getdeploying.com/gpus/nvidia-a100`, 2026. accessed June 2026.

[7] Beck Taylor, Catherine Henshall, Sara Kenyon, Ian Litchfield, and Sheila Greenfield. Can rapid approaches to qualitative analysis deliver timely, valid findings to clinical leaders? A mixed methods study comparing rapid and thematic analysis. *BMJ Open*, 8(10):e019993, 2018. doi: 10.1136/bmjopen-2017-019993.
